# ADHD Presentations and Severity in Adults: Psychometric Profiles, Psychiatric Comorbidities, and Emotional Dysregulation in Relation to Psychiatric Hospitalization

**DOI:** 10.3390/jcm15207953

**Published:** 2026-10-14

**Authors:** Miriam Olivola, Paola Landi, Vassilis Martiadis, Arianna De Ciechi, Chiara Bucca, Yacob Levin Reibman, Luca Giacovelli, Elena Bernoni, Viviana Venturi, Bernardo Dell’Osso

**Affiliations:** 1 Department of Mental Health and Addiction Services, Azienda Socio Sanitaria Territoriale (ASST) Fatebenefratelli-Sacco, 20100 Milan, Italy; paola.landi@asst-fbf-sacco.it (P.L.); bucca.chiara@asst-fbf-sacco.it (C.B.); yacob.reibman@asst-fbf-sacco.it (Y.L.R.); giacovelli.luca@asst-fbf-sacco.it (L.G.); elena.bernoni@asst-fbf-sacco.it (E.B.); viviana.venturi@asst-fbf-sacco.it (V.V.); dellosso.bernardo@asst-fbf-sacco.it (B.D.); 2 Department of Brain and Behavioral Sciences, University of Pavia, 27100 Pavia, Italy; 3 Department of Mental Health, Azienda Sanitaria Locale Napoli 1 Centro, 80125 Naples, Italy; vassilis.martiadis@aslnapoli1centro.it; 4 Department of Philosophy, Social Sciences and Education, University of Perugia, 06123 Perugia, Italy; arianna.deciechi@dottorandi.unipg.it; 5 Department of Biomedical and Clinical Sciences “Luigi Sacco”, University of Milan, 20157 Milan, Italy; 6 “Aldo Ravelli” Center for Nanotechnology and Neurostimulation, University of Milan, 20143 Milan, Italy; 7 Department of Psychiatry and Behavioral Sciences, Stanford University, Stanford, CA 94305, USA; 8 Centro per lo Studio dei Meccanismi Molecolari alla Base delle Patologie Neuro-Psico-Geria-Triche, University of Milan, 20157 Milan, Italy

**Keywords:** ADHD, adults, emotional dysregulation, psychiatric comorbidity, substance use disorder, hospitalization

## Abstract

**Background/Objectives:** Attention-deficit/hyperactivity disorder (ADHD) in adults is heterogeneous in symptom presentation, severity, and comorbid psychopathology, yet systematic data from Italian clinical populations remain limited. **Methods:** In this retrospective, cross-sectional study, we analyzed routine clinical data from 219 adults with a DSM-5 diagnosis of ADHD at a specialist mental health service in Milan. We compared psychometric profiles and psychiatric comorbidities across ADHD presentations (Combined vs. Inattentive), examined the associations of clinician-rated ADHD severity with dimensional and categorical outcomes, and explored, in an exploratory cross-sectional indirect-effect analysis, whether emotional dysregulation statistically accounted for the association between ADHD severity and psychiatric hospitalization. Multiplicity was addressed with Holm and false-discovery-rate procedures within predefined families of outcomes. Measures included the Zung Depression Scale, STAI, Difficulties in Emotion Regulation Scale (DERS), Internet Addiction Test, Adult ADHD Quality of Life Scale, Barratt Impulsiveness Scale, and the SCID-5-PD. Analyses comprised MANCOVA, multiple linear and binary logistic regression, and an exploratory indirect-effect analysis (PROCESS Model 4). **Results:** The Combined presentation was associated with nearly fivefold higher odds of substance use disorder than the Inattentive presentation (robust to Holm correction), whereas the overall multivariate difference in psychometric profiles was not significant and the higher emotional dysregulation in the Combined presentation was a small effect not robust to multiplicity correction. Greater ADHD severity was associated with greater emotional dysregulation and higher odds of substance use disorder (both robust to Holm correction) and, less robustly, with depressive symptoms, problematic internet use, and lower quality of life. A statistically significant cross-sectional indirect association between ADHD severity and hospitalization via emotional dysregulation was observed (indirect effect B = 0.21, 95% CI [0.03, 0.50]); because the temporal ordering of these variables could not be established, this finding does not demonstrate mediation. Female sex was independently associated with lower quality of life and higher depressive symptoms, and higher educational level was associated with lower odds of substance use disorder and hospitalization. **Conclusions:** These findings, obtained in a specialist clinical sample, support the clinical relevance of assessing ADHD presentation, severity, and emotional dysregulation, and generate hypotheses that require confirmation in prospective, multicentre studies.

## 1. Introduction

Attention-deficit/hyperactivity disorder (ADHD) is one of the most prevalent neurodevelopmental disorders, with adult prevalence estimates that vary substantially according to age, case definition, persistence of childhood-onset symptoms, and ascertainment method: pooled estimates are approximately 2.5–2.6% for persistent ADHD with childhood onset and up to 6.8% for symptomatic adult ADHD irrespective of childhood onset [1,2,3] with further variation across register-based, survey, and clinical studies [4,5]. Although historically conceptualized as a childhood-onset condition, longitudinal evidence consistently demonstrates that ADHD persists into adulthood in a substantial proportion of individuals, often with significant functional impairment across occupational, social, and academic domains [6,7,8].

According to the DSM-5, ADHD is classified into three presentations: Predominantly Inattentive (ADHD-I), Predominantly Hyperactive-Impulsive (ADHD-HI), and Combined (ADHD-C) [9]. In adult clinical populations, the Combined and Inattentive presentations are the most frequently encountered, while the Hyperactive-Impulsive presentation is comparatively rare [10,11]. DSM-5 also allows clinicians to specify current severity (mild, moderate, or severe) on the basis of the number of symptoms in excess of those required for the diagnosis and the degree of functional impairment, thus providing a dimensional complement to the categorical presentation. The differential clinical profile of these presentations—particularly regarding associated psychopathology and functional outcomes—has been a subject of ongoing investigation.

Psychiatric comorbidity is the rule rather than the exception in adult ADHD. Registry-based data from large European samples indicate that over 60% of adults newly diagnosed with ADHD present with at least one comorbid psychiatric condition [12]. most frequently observed comorbidities include substance use disorders (SUD), anxiety disorders, and mood disorders [12,13]. The Combined presentation has generally been associated with higher rates of externalizing comorbidities, including SUD and conduct-related problems, while the Inattentive presentation has been more variably linked to internalizing disorders such as depression and anxiety [11,14]. However, findings across studies remain inconsistent, likely reflecting differences in sample characteristics, assessment methods, and healthcare settings [15].

Beyond categorical comorbidities, adults with ADHD frequently present with clinically significant emotional dysregulation (ED), defined as difficulties in modulating the intensity and duration of emotional responses. Estimates of ED prevalence in adult ADHD range from 34% to 70% depending on the operationalization used [16]. ED has been identified as a transdiagnostic dimension that co-occurs with and amplifies core ADHD symptomatology [17,18], and is associated with greater symptom severity, higher rates of comorbid depression, worse interpersonal functioning, and reduced quality of life [19,20]. Whether ED is associated with more severe clinical outcomes, such as psychiatric hospitalization, in adults with ADHD has received limited empirical attention; cross-sectional data can describe whether such associations exist, whereas prospective designs are required to establish their temporal ordering.

Problematic internet use (PIU) and internet addiction (IA) represent an increasingly recognized behavioral correlate of adult ADHD. Meta-analytic evidence confirms a moderate positive association between ADHD and IA [21], with longitudinal data suggesting that ADHD symptom severity—particularly motivational dysfunction—predicts the development of internet addiction over time [22]. Hyperfocus, a feature often associated with ADHD, has been identified as a significant mediator of this relationship [23]. Clinically, IA in adults with ADHD is associated with greater impulsiveness and poorer quality of life [24,25].

Quality of life (QoL) is consistently impaired in adults with ADHD relative to the general population [26,27]. Both symptom severity and comorbid psychopathology—particularly depression and ED—independently contribute to reduced QoL [28,29]. Sex differences in QoL have also been documented, with female adults with ADHD often reporting greater functional impairment and higher rates of internalizing comorbidities [16,20].

Despite this growing body of evidence, data from Italian specialist mental health services remain scarce. The present study addresses this gap through a retrospective analysis of routinely collected clinical data from an adult ADHD sample followed at the Department of Mental Health, ASST Fatebenefratelli Sacco, Milan, examining (1) the psychometric and comorbidity profiles associated with ADHD presentation (Combined vs. Inattentive); (2) the associations between clinician-rated ADHD severity and a broad range of dimensional and categorical outcomes; and (3) in an exploratory cross-sectional analysis, whether emotional dysregulation statistically accounted for the association between ADHD severity and psychiatric hospitalization. To address the number of statistical tests, analyses were organized into primary and secondary/exploratory families of outcomes (Section 2.4).

## 2. Materials and Methods

### 2.1. Study Design and Setting

This was a retrospective, cross-sectional study based on clinical data routinely collected at the Department of Mental Health, ASST Fatebenefratelli Sacco, Milan, Italy. Data were extracted from the clinical records of adults consecutively referred to the outpatient ADHD service between 2020 and 2023, in whom the measures had been administered as part of routine care. Because the study was a retrospective analysis of anonymized, routinely collected data, formal ethics committee approval is generally not required, since the study involved nothing beyond routine clinical practice; nevertheless, the authors drafted a study protocol (ADHD-YA, version 1.0, 8 August 2026), which has been submitted to the Comitato Etico Territoriale Lombardia 1 (opinion pending), and prepared a Data Protection Impact Assessment (DPIA) pursuant to Article 35 of the GDPR. The study followed the Declaration of Helsinki. The study is reported in accordance with the STROBE (Strengthening the Reporting of Observational Studies in Epidemiology) guidelines for cross-sectional studies [30]; the completed STROBE checklist is provided as Supplementary Material (Table S35).

### 2.2. Participants

The sample comprised *N* = 219 adults with a confirmed DSM-5 diagnosis of ADHD. Inclusion criteria were: (1) age ≥ 18 years; (2) confirmed ADHD diagnosis according to DSM-5 criteria, established through the diagnostic procedure described below; (3) sufficient linguistic competence to complete self-report measures. Exclusion criteria included: active psychosis, severe intellectual disability, or inability to provide informed consent.

The sample included all adults who regularly attended the outpatient ADHD service during the study period and received a DSM-5 diagnosis of ADHD confirmed with the DIVA-5 at our centre (*N* = 219); no further exclusions were applied. The number of participants contributing to each analysis is reported in Table 1 and in the tables of results.

ADHD diagnostic procedure. ADHD was diagnosed by psychiatrists experienced in adult ADHD according to DSM-5 criteria, using the Diagnostic Interview for ADHD in Adults (DIVA-5; [31]), a semi-structured interview that systematically assesses each of the 18 DSM-5 ADHD symptoms in childhood and adulthood and documents impairment across work/education, relationships, social life, leisure, and self-confidence. Childhood onset of symptoms was established retrospectively through the childhood section of the DIVA, supplemented by the Wender Utah Rating Scale (WURS; [32]) and, where available, by collateral information from parents or other relatives and by school reports. Functional impairment was established on the basis of the DIVA impairment sections and the clinical interview. ADHD presentation (Combined, Predominantly Inattentive, or Predominantly Hyperactive-Impulsive) was assigned according to the DSM-5 symptom-count criteria for the past six months. The same diagnostic procedure was applied throughout the recruitment period.

ADHD presentations were classified as Combined (*n* = 168), Inattentive (*n* = 48), or Hyperactive-Impulsive (*n* = 3). Due to the very small number of participants with the Predominantly Hyperactive-Impulsive presentation (*n* = 3), this group was excluded from all inferential analyses involving ADHD presentation; these participants were retained in the analyses of ADHD severity. Consequently, comparisons between presentations were conducted between the Combined and Inattentive presentations only, and no conclusions can be drawn regarding the Predominantly Hyperactive-Impulsive presentation.

### 2.3. Measures

ADHD Severity. Current ADHD severity was rated by the assessing psychiatrist at the end of the diagnostic evaluation using the DSM-5 severity specifier [9]: mild (few, if any, symptoms in excess of those required to make the diagnosis, and symptoms resulting in no more than minor functional impairment), moderate (symptoms or functional impairment between mild and severe), and severe (many symptoms in excess of those required, several particularly severe symptoms, or marked functional impairment). Severity was derived from the DIVA-5 assessment, integrating the number of current symptoms and the functional impairment documented across domains. Severity was coded 1 = mild, 2 = moderate, and 3 = severe; 28 (12.8%), 133 (60.7%), and 58 (26.5%) participants were rated as mild, moderate, and severe, respectively (Table 1 and Table 2). Ratings were made by two experienced clinicians; inter-rater reliability was not formally assessed.

Zung Self-Rating Depression Scale (ZUNG). A 20-item self-report measure of depressive symptom severity, with higher scores indicating greater depression [33]. Scores were analyzed as a continuous variable, using the raw total score (sum of the 20 items; range 20–80) throughout.

State-Trait Anxiety Inventory (STAI). A validated 40-item self-report measure assessing state (STAI-S) and trait (STAI-T) anxiety [34]. Both subscales were analyzed as continuous outcomes.

Difficulties in Emotion Regulation Scale (DERS). A 36-item self-report questionnaire assessing multiple dimensions of emotional dysregulation, including non-acceptance of emotional responses, difficulties engaging in goal-directed behavior, impulse control difficulties, lack of emotional awareness, limited access to emotion regulation strategies, and lack of emotional clarity [35]. Total scores range from 36 to 180, with higher scores indicating greater dysregulation.

Internet Addiction Test (IAT). A 20-item self-report scale assessing the severity of problematic internet use [36]. Total scores range from 20 to 100.

Adult ADHD Quality of Life Scale (AAQoL). A validated 29-item self-report measure of health-related quality of life specifically designed for adults with ADHD [37]. Higher scores indicate better quality of life.

Barratt Impulsiveness Scale (BIS-11). A 30-item self-report measure of impulsivity [38]. The BIS-11 total score was used only as one of the psychometric correlates in the secondary multivariable model of psychiatric hospitalization (Section 2.4).

Psychiatric Comorbidities. Mood disorders, anxiety disorders, substance use disorders, and other psychiatric conditions were diagnosed by the assessing psychiatrists according to DSM-5 criteria, integrating the clinical interview and records with the psychometric assessment: the ZUNG for depressive disorders, the Mood Disorder Questionnaire (MDQ; [39]) for bipolar spectrum disorders, and the STAI for anxiety disorders; personality disorders were assessed with the Structured Clinical Interview for DSM-5 Personality Disorders (SCID-5-PD; [40]. Italian edition). Psychiatric and personality disorder diagnoses referred to the lifetime period, whereas SUD referred to a substance use disorder that was current at the index assessment; a past history of substance use and the type of substance involved (stimulants, opioids, sedatives, cannabinoids, ecstasy, or novel psychoactive substances) were recorded separately. Because autism spectrum disorder (ASD) requires a dedicated assessment, ASD diagnoses had either been made previously by private specialist centres or were established through a structured assessment carried out by a dedicated team at our Department of Mental Health, according to DSM-5 criteria. Diagnoses were coded as binary variables (0 = absent, 1 = present).

**Table 2:** Multiplicity-Adjusted *p* Values Within Predefined Families of Outcomes.

Family/Outcome	Nominal *p*	Holm-Adjusted *p*	FDR q	Robust to Holm
**1. Presentation: psychometric outcomes (ANCOVA; MANCOVA omnibus *p* = 0.068)**				
ZUNG (total score)	0.081	0.324	0.203	No
STAI-State	0.853	1.000	0.856	No
STAI-Trait	0.856	1.000	0.856	No
DERS	0.028	0.140	0.140	No
IAT	0.299	0.897	0.498	No
**2. Presentation: psychiatric comorbidities (logistic regression)**				
Substance use disorder	0.006	0.028	0.028	Yes
Mood disorder	0.502	1.000	0.567	No
Anxiety disorder	0.567	1.000	0.567	No
Autism spectrum disorder	0.123	0.492	0.308	No
Other psychiatric disorders	0.190	0.570	0.317	No
**3. Severity: psychometric outcomes (linear regression)**				
DERS	<0.001	0.003	0.003	Yes
ZUNG	0.018	0.073	0.030	No (FDR only)
STAI-State	0.095	0.095	0.095	No
STAI-Trait	0.046	0.092	0.055	No
IAT	0.014	0.072	0.030	No (FDR only)
AAQoL	0.020	0.073	0.030	No (FDR only)
**4. Severity: psychiatric comorbidities (logistic regression)**				
Substance use disorder	0.002	0.010	0.010	Yes
Anxiety disorder (inverse)	0.025	0.127	0.076	No
Mood disorder	0.066	0.262	0.131	No
Sleep disturbance	0.233	0.467	0.280	No
Other psychiatric disorders	0.118	0.353	0.176	No
Autism spectrum disorder	0.290	0.467	0.290	No
**5. Indirect-effect model (single test)**				
Severity → DERS → hospitalization	95% CI [0.03, 0.50]	—	—	Not adjusted (single test)

Note. Holm-adjusted *p* values control the family-wise error rate (Holm, 1979 [41]); FDR q values are Benjamini–Hochberg adjusted (Benjamini & Hochberg, 1995 [42]). Nominal *p* values were recomputed from the reported test statistics (t or Wald χ^2^) where available. Secondary/exploratory analyses (family history, organic comorbidity, treatment status, DUI, and hospitalization models) were not adjusted.

Socio-Demographic Variables. Age, sex (self-reported as male or female; 0 = male, 1 = female), and educational level (coded as an ordinal variable) were collected at the time of clinical assessment.

Duration of Untreated Illness (DUI). Defined as the time (in years) between the diagnosis of ADHD and the initiation of any ADHD treatment. DUI was available for 97 participants because the exact age at diagnosis could not be retrieved for the remaining participants.

Psychiatric Hospitalization. Recorded as a binary variable (0 = no, 1 = yes) indicating any lifetime admission to a psychiatric inpatient unit, as documented in the clinical record. Because the dates of admission were not available, it could not be established whether admissions preceded or followed the index ADHD assessment. Overall, 32 participants (14.6%) had a history of psychiatric hospitalization (Table 1 and Table 2).

Treatment status and timing of assessments. Current pharmacological treatment and current psychotherapy were recorded at the first visit (index assessment). All self-report questionnaires were administered during the diagnostic work-up at the index assessment, i.e., before the initiation of ADHD-specific treatment at our service; some participants may, however, have been receiving other psychiatric treatments at that time. Questionnaires were completed in the clinic as part of the routine assessment; no additional research-specific quality-control procedures were applied.

### 2.4. Statistical Analyses

All analyses were conducted using IBM SPSS Statistics (version 28.0). The significance threshold was set at α = 0.05 (two-tailed). Effect sizes were reported as partial η^2^ for ANOVA-based analyses and Nagelkerke R^2^ for logistic regression models. Model assumptions were evaluated prior to estimation. For linear models, linearity, normality of residuals, homoscedasticity, and multicollinearity were examined. For logistic regression models, multicollinearity and overall model fit were assessed using the Hosmer-Lemeshow goodness-of-fit test. Missing data were handled by complete-case analysis; the number of participants with available data for each variable is reported in Table 1 and Table 2, and participants with complete versus incomplete psychometric data were compared on age, sex, educational level, ADHD presentation, and ADHD severity using independent-samples *t* tests and Pearson chi-square tests (Table S36). Because all models were adjusted for sex, any difference in sex distribution between these groups was partly accounted for in the analyses.

Inferential hierarchy and multiplicity. To limit Type I error inflation, analyses were organized into families of outcomes. Primary analyses were: (i) the MANCOVA comparing presentations across the five psychometric outcomes, followed by univariate ANCOVAs; (ii) the logistic regressions comparing presentations on the five psychiatric comorbidity outcomes (mood, anxiety, substance use, autism spectrum, and other psychiatric disorders); (iii) the linear regressions of ADHD severity on the six psychometric outcomes (DERS, ZUNG, STAI-State, STAI-Trait, IAT, and AAQoL); (iv) the logistic regressions of ADHD severity on the six psychiatric comorbidity outcomes (the five listed above plus sleep disturbance); and (v) the indirect-effect model. Within each family, *p* values were adjusted using the Holm procedure [41], which controls the family-wise error rate; the Benjamini–Hochberg false-discovery-rate (FDR) procedure [42] was reported as a less conservative sensitivity analysis (Table 2) All other analyses—comparisons involving family history, organic comorbidity, treatment status, DUI, quality of life by presentation, and the multivariable hospitalization models—were considered secondary and exploratory, were not adjusted for multiplicity, and are interpreted on the basis of effect sizes and confidence intervals rather than nominal *p* values. Results that did not remain significant after Holm correction are explicitly described as exploratory.

Comparisons between presentations. A multivariate analysis of covariance (MANCOVA) was conducted to examine whether ADHD presentation (Combined vs. Inattentive) was associated with differences across five psychometric outcomes (ZUNG, STAI-State, STAI-Trait, DERS total, IAT total), adjusting for age, sex, and educational level. Follow-up univariate ANCOVAs were conducted for each dependent variable. Differences in socio-demographic characteristics by ADHD presentation were examined using ANCOVA (age) and chi-square tests (sex and education). Binary logistic regression models were used to examine the association between ADHD presentation and categorical comorbidity outcomes, adjusting for age, sex, and educational level.

Severity analyses. ADHD severity was coded according to increasing levels of clinical severity (1 = mild, 2 = moderate, 3 = severe) and entered into regression models as a single ordinal predictor, which assumes an approximately linear effect with equal spacing between adjacent categories. This assumption was examined in sensitivity analyses in which severity was dummy-coded (mild as reference) and a test of linear trend was performed; the same approach was applied to educational level, which was entered as an ordinal covariate (low education as reference). Sensitivity analyses were performed for the two associations that remained significant after multiplicity correction (DERS and SUD) and were adjusted for age, sex, and, respectively, educational level or ADHD severity (Table S37). Multiple linear regression models were used to examine the associations between ADHD severity and continuous outcomes (DERS, ZUNG, STAI-State, STAI-Trait, IAT, AAQoL, DUI), adjusting for age, sex, and educational level. Binary logistic regression models were used to examine the associations between ADHD severity and categorical comorbidity outcomes (SUD, mood disorder, anxiety disorder, sleep disturbance, other psychiatric disorders, autism spectrum disorder) and, as secondary exploratory variables, organic comorbidity, family psychiatric history, family ADHD history, current pharmacological treatment, and current psychotherapy, adjusting for age, sex, and educational level. The term “predictor” is used in the technical regression sense only and does not imply temporal precedence; in particular, family history variables cannot be considered outcomes of the proband’s ADHD severity.

Hospitalization models. As secondary, exploratory analyses, two multivariable logistic models were estimated for psychiatric hospitalization: (a) a model including the psychometric measures (DERS, ZUNG, STAI-State, IAT, and BIS-11), selected a priori as indices of the emotional, affective, behavioural, and impulsive dimensions most often linked to severe psychiatric outcomes in the literature, together with age, sex, and educational level; and (b) a model including ADHD severity and presentation simultaneously, adjusted for the same covariates. Because the number of events relative to the number of parameters was limited (*N* = 172; 24 events; 3.0 events per predictor), model (a) was re-estimated using Firth’s penalized-likelihood logistic regression to reduce small-sample and sparse-data bias [43,44] (Table S38).

Exploratory indirect-effect analysis. An exploratory cross-sectional indirect-effect analysis was conducted using the PROCESS macro (Model 4; [45]), with 5000 bootstrap samples, to estimate whether emotional dysregulation (DERS total) statistically accounted for part of the association between ADHD severity and psychiatric hospitalization, controlling for age, sex, and educational level. Indirect effects were considered significant when the 95% bootstrap confidence interval did not include zero. Because ADHD severity and DERS were measured at the same assessment and hospitalization refers to lifetime admissions whose timing relative to this assessment is unknown, the model tests a pattern of cross-sectional statistical associations only; it does not establish temporal or causal ordering and cannot be interpreted as a test of mediation [46,47].

## 3. Results

### 3.1. Sample Characteristics and Comparability of Presentations

The socio-demographic and clinical characteristics of the sample, including the number of participants with available data for each variable, are reported in Table 1 and Table 2. Overall, 57 participants (26.0%) had a current SUD, and 32 (14.6%) had a history of psychiatric hospitalization. Participants with complete (*n* = 86) and incomplete (*n* = 133) psychometric data did not differ significantly in age, t(217) = −0.31, *p* = 0.759, educational level, χ^2^(2) = 0.85, *p* = 0.654, ADHD presentation, χ^2^(1) = 0.04, *p* = 0.852, or ADHD severity, χ^2^(2) = 1.55, *p* = 0.460. However, sex distribution differed significantly, χ^2^(1) = 11.63, *p* = 0.001: women represented a greater proportion of participants with complete than of those with incomplete psychometric data (56.5% vs. 33.1%; Table S36).

No significant differences in socio-demographic characteristics emerged between the Combined and Inattentive presentations (Table S18). An ANCOVA indicated that age did not differ significantly between presentations, F(1, 211) = 0.34, *p* = 0.559, partial η^2^ = 0.002. ADHD presentation was not significantly associated with sex distribution, χ^2^(1) = 0.48, *p* = 0.487, Cramer’s V = 0.047, nor with educational level, χ^2^(3) = 4.70, *p* = 0.195, Cramer’s V = 0.148. The two groups were thus comparable in terms of age, sex, and educational attainment, supporting the comparability of the two presentations in subsequent analyses.

### 3.2. Psychometric Profiles by ADHD Presentation

A MANCOVA was conducted to examine whether ADHD presentation was associated with differences in psychometric functioning, adjusting for age, sex, and educational level. Using the ZUNG raw total score, the overall multivariate effect of ADHD presentation did not reach statistical significance, Wilks’ Λ = 0.939, F(5, 162) = 2.10, *p* = 0.068, partial η^2^ = 0.061 (Table 3).

Sex showed a significant multivariate effect, Wilks’ Λ = 0.929, F(5, 162) = 2.47, *p* = 0.034, partial η^2^ = 0.071, whereas age and education did not reach statistical significance.

In exploratory follow-up univariate ANCOVAs, individuals with the Combined presentation reported greater emotional dysregulation (DERS total) than those with the Inattentive presentation, F(1, 166) = 4.93, *p* = 0.028, partial η^2^ = 0.029 (adjusted means: 117.00, SE = 2.07, vs. 106.50, SE = 4.24), whereas the difference in depressive symptoms (ZUNG total score) did not reach statistical significance, F(1, 166) = 3.09, *p* = 0.081, partial η^2^ = 0.018 (adjusted means: 48.63, SE = 0.91, vs. 44.99, SE = 1.86) (Table 4; Table S1). No significant differences emerged for state anxiety, trait anxiety, or problematic internet use. The univariate difference in DERS was small (partial η^2^ = 0.029) and did not remain significant after Holm correction within the family of five psychometric outcomes (adjusted *p* = 0.140; FDR q = 0.140; Table 2); it should therefore be regarded as exploratory.

### 3.3. Psychiatric Comorbidities by ADHD Presentation

Binary logistic regression analyses examined the associations between ADHD presentation and specific psychiatric comorbidities, adjusting for age, sex, and educational level.

Mood disorders. The overall model was not statistically significant, χ^2^(4) = 1.09, *p* = 0.897 (Cox & Snell R^2^ = 0.005). ADHD presentation was not a significant predictor of mood disorder comorbidity, B = −0.290, SE = 0.433, *p* = 0.502, OR = 0.75, 95% CI [0.32, 1.75] (Table S2).

Anxiety disorders. The overall model was statistically significant, χ^2^(4) = 11.78, *p* = 0.019 (Cox & Snell R^2^ = 0.053). ADHD presentation was not a significant predictor of anxiety disorder comorbidity, B = −0.207, SE = 0.361, *p* = 0.567, OR = 0.81, 95% CI [0.40, 1.65] (Table S3). Educational level emerged as the only significant predictor, B = 0.721, SE = 0.269, *p* = 0.007, OR = 2.06, 95% CI [1.21, 3.48].

Substance use disorder. The overall model was statistically significant, χ^2^(4) = 33.61, *p* < 0.001 (Cox & Snell R^2^ = 0.145). ADHD presentation was a significant predictor of SUD, B = 1.566, SE = 0.565, *p* = 0.006, OR = 4.79, 95% CI [1.58, 14.49], indicating nearly five times higher odds of SUD in the Combined than the Inattentive presentation (Table 5). SUD was present in 51 of 168 participants (30.4%) with the Combined presentation and in 4 of 48 (8.3%) with the Inattentive presentation; among participants with SUD, the substance of problematic use or dependence was most frequently stimulants (36/57, 63.2%), followed by novel psychoactive substances (13/57, 22.8%), ecstasy (7/57, 12.3%), opioids (3/57, 5.3%), and cannabinoids (1/57, 1.8%; categories not mutually exclusive; Table 1 and Table 2). This association remained significant after Holm correction across the five psychiatric comorbidity outcomes (adjusted *p* = 0.028; Table 2). Educational level was also significantly associated with SUD, B = −1.033, SE = 0.278, *p* < 0.001, OR = 0.36, 95% CI [0.21, 0.61], with higher education associated with lower odds of SUD.

Other psychiatric disorders. The overall model did not reach statistical significance, χ^2^(4) = 8.21, *p* = 0.084 (Cox & Snell R^2^ = 0.037). Age was the only significant predictor, B = 0.103, SE = 0.050, *p* = 0.040, OR = 1.11, 95% CI [1.01, 1.22] (Table S4).

Autism spectrum disorder. The overall model did not reach statistical significance, χ^2^(4) = 8.55, *p* = 0.073 (Cox & Snell R^2^ = 0.039). ADHD presentation was not significantly associated with autism spectrum comorbidity, B = 1.615, SE = 1.046, *p* = 0.123, OR = 5.03, 95% CI [0.65, 39.07]; the wide confidence interval reflects the relatively low prevalence of autism spectrum disorder in the sample (8.2%) and the limited number of ASD cases (Table S5).

The following clinical variables (family psychiatric and ADHD history, organic comorbidity, and current treatment) were examined as secondary, exploratory outcomes and were not corrected for multiplicity.

Family psychiatric history. ADHD presentation was not significantly associated with family psychiatric history, OR = 1.69, *p* = 0.117 (Table S19). The overall model was not significant, χ^2^(4) = 5.00, *p* = 0.287.

Family ADHD history. ADHD presentation was not associated with family ADHD history (*p* = 0.606), while female sex was strongly associated with higher odds of reporting family ADHD history, OR = 5.45, *p* < 0.001 (Table S21).

Organic comorbidity. ADHD presentation was not associated with organic comorbidity (*p* = 0.961). Female sex was independently associated with higher odds of organic comorbidity, OR = 2.24, *p* = 0.009 (Table S25). The presentation × sex interaction was not significant (*p* = 0.440; Table S27).

Current pharmacological treatment. ADHD presentation was not a significant predictor of current pharmacological treatment, OR = 1.75, *p* = 0.100 (Table S33). Educational level emerged as the only significant predictor, OR = 0.61, *p* = 0.045, indicating that higher education was associated with lower odds of receiving pharmacological treatment.

Current psychotherapy. Neither ADHD presentation nor any covariate was significantly associated with current psychotherapy engagement (Table S23).

### 3.4. Associations Between ADHD Severity and Dimensional Outcomes

Separate multiple linear regression models examined the associations between ADHD severity and continuous psychometric outcomes, adjusting for age, sex, and educational level (Table 6).

Emotional dysregulation. The overall model was statistically significant, F(4, 203) = 3.60, *p* = 0.007, R^2^ = 0.066. ADHD severity was a significant positive predictor of DERS scores, B = 9.55, SE = 2.68, β = 0.248, t = 3.56, *p* < 0.001 (Table S6).

Depressive symptoms. The overall model was statistically significant, F(4, 186) = 4.64, *p* = 0.001, R^2^ = 0.091. ADHD severity was a significant positive predictor of ZUNG scores, B = 2.96, SE = 1.24, β = 0.171, t = 2.38, *p* = 0.018 (Table S7). Female sex was also a significant predictor, B = 4.999, SE = 1.59, β = 0.226, t = 3.15, *p* = 0.002, with females reporting higher depressive symptoms.

Trait anxiety. The overall model was not statistically significant, F(4, 188) = 2.36, *p* = 0.055, explaining 4.8% of the variance in trait anxiety (Adjusted R^2^ = 0.028). Nevertheless, ADHD severity emerged as a significant positive predictor of trait anxiety, B = 2.39, SE = 1.19, β = 0.147, t = 2.01, *p* = 0.046. Sex showed a trend-level association, B = 2.53, SE = 1.51, *p* = 0.097, while age and education were not significant predictors (Table S9).

State anxiety. The overall model was not statistically significant, F(4, 188) = 1.90, *p* = 0.112. ADHD severity was not a significant predictor of state anxiety (*p* = 0.095; Table S8).

Problematic internet use. The overall model was statistically significant, F(4, 195) = 2.70, *p* = 0.032, R^2^ = 0.053. ADHD severity was a significant positive predictor of IAT scores, B = 4.55, SE = 1.84, β = 0.176, t = 2.47, *p* = 0.014 (Table S10).

Quality of life. The overall model was statistically significant, F(4, 192) = 5.46, *p* < 0.001, R^2^ = 0.102. Higher ADHD severity was significantly associated with lower AAQoL scores, B = −4.17, SE = 1.78, β = −0.163, *p* = 0.020 (Table S29). Female sex was also independently associated with lower quality of life, B = −6.70, *p* = 0.002.

Duration of untreated illness. The overall model was statistically significant, F(4, 92) = 37.90, *p* < 0.001, R^2^ = 0.622; however, this effect was almost entirely driven by age (β = 0.798, *p* < 0.001), and DUI was available for fewer than half of the sample (*n* = 97). ADHD severity was not associated with DUI (*p* = 0.910; Table S12).

After multiplicity correction within the family of six psychometric outcomes, the association between ADHD severity and DERS remained significant under the Holm procedure (adjusted *p* = 0.003). The associations with ZUNG, IAT, and AAQoL remained significant under FDR control (q = 0.030) but not under the Holm procedure (adjusted *p* = 0.072–0.073), whereas the association with trait anxiety did not survive either procedure (Holm-adjusted *p* = 0.092; q = 0.055) (Table 2). Sensitivity analyses supported the ordinal specification of severity for DERS (Table S37): the linear trend across severity levels remained significant, B = 9.44, SE = 2.69, β = 0.245, *p* = 0.001; with dummy coding (mild as reference), the moderate group did not differ significantly from the mild group, B = 3.50, SE = 4.95, *p* = 0.480, whereas the severe group reported significantly greater emotional dysregulation, B = 16.82, SE = 5.56, *p* = 0.003. Educational level was not associated with DERS under either ordinal or dummy coding (all ps ≥ 0.126).

### 3.5. Associations Between ADHD Severity and Categorical Outcomes

Binary logistic regression analyses examined the associations between ADHD severity and categorical comorbidity outcomes, adjusting for age, sex, and educational level (Table 7).

Substance use disorder. ADHD severity was a significant positive predictor of SUD, B = 0.93, SE = 0.30, *p* = 0.002, OR = 2.54, 95% CI [1.42, 4.54] (Table S13; Nagelkerke R^2^ = 0.202). Under the linear coding of severity, each one-level increase in ADHD severity was associated with more than double the odds of SUD. This association remained significant after Holm correction across the six comorbidity outcomes (adjusted *p* = 0.010; Table 2). In sensitivity analyses (Table S37), the linear severity trend for SUD remained significant, OR = 2.50, 95% CI [1.40, 4.46], *p* = 0.002; with dummy coding, the moderate group did not differ significantly from the mild group, OR = 1.91, 95% CI [0.52, 7.09], *p* = 0.331, whereas the severe group had higher odds of SUD, OR = 5.24, 95% CI [1.35, 20.41], *p* = 0.017. The inverse association between educational level and SUD was retained under dummy coding (high school vs. low education: OR = 0.26, 95% CI [0.11, 0.61], *p* = 0.002; university vs. low education: OR = 0.17, 95% CI [0.06, 0.50], *p* = 0.001). Educational level was inversely associated with SUD, OR = 0.41, *p* = 0.001.

Anxiety disorder. ADHD severity was significantly associated with anxiety disorder, B = −0.594, SE = 0.265, *p* = 0.025, OR = 0.55, 95% CI [0.33, 0.93] (Table S15), indicating that higher ADHD severity was unexpectedly associated with lower odds of an anxiety disorder diagnosis. This association did not survive multiplicity correction (Holm-adjusted *p* = 0.127; FDR q = 0.076) and is therefore regarded as exploratory. Educational level was a significant positive predictor, OR = 1.95, *p* = 0.015.

Mood disorder. ADHD severity showed a trend-level association with mood disorder, OR = 1.82, *p* = 0.066, which did not reach statistical significance (Table S14).

Sleep disturbance. ADHD severity was not significantly associated with sleep disturbance, OR = 1.37, *p* = 0.233 (Table S11). Education showed a trend-level inverse association (*p* = 0.054).

Other psychiatric disorders. ADHD severity was not a significant predictor, OR = 1.90, *p* = 0.118. Age showed a borderline effect, OR = 1.10, *p* = 0.050 (Table S16).

Autism spectrum disorder. ADHD severity was not significantly associated with autism spectrum disorder, OR = 0.65, *p* = 0.290. Educational level was a significant negative predictor, OR = 0.44, *p* = 0.038 (Table S17).

Family psychiatric history. Higher ADHD severity was associated with increased odds of a positive family psychiatric history, OR = 1.90, *p* = 0.008 (Table S20; Nagelkerke R^2^ = 0.06). No other variables were significant. Because family history cannot be an outcome of the proband’s current severity, this exploratory association is reported as a non-directional correlate only.

Family ADHD history. ADHD severity was not associated with family ADHD history (*p* = 0.976). Female sex remained a strong predictor, OR = 5.47, *p* < 0.001 (Table S22).

Organic comorbidity. ADHD severity was not associated with organic comorbidity (*p* = 0.658). Female sex was a significant predictor, OR = 2.21, *p* = 0.010 (Table S26).

Current pharmacological treatment. ADHD severity was not significantly associated with current pharmacological treatment, OR = 1.36, *p* = 0.201 (Table S34). Educational level showed a trend-level inverse association (*p* = 0.074).

Current psychotherapy. ADHD severity was not significantly associated with current psychotherapy engagement (Table S24).

### 3.6. Quality of Life by ADHD Presentation

An ANCOVA examined whether ADHD presentation was associated with quality of life (AAQoL), controlling for age, sex, and educational level. The overall model was significant, F(4, 189) = 3.66, *p* = 0.007, adjusted R^2^ = 0.052. ADHD presentation was not significantly associated with AAQoL, F(1, 189) = 0.49, *p* = 0.485, partial η^2^ = 0.003 (Table S28). Female sex emerged as a significant predictor, F(1, 189) = 8.85, *p* = 0.003, partial η^2^ = 0.045, with females reporting lower quality of life.

### 3.7. Hospitalization: Correlates and Exploratory Indirect-Effect Analysis

Overall, 32 of 219 participants (14.6%) had a history of psychiatric hospitalization. In the secondary, exploratory model including psychometric measures (*N* = 172; 24 hospitalization events and 148 non-events; 3.0 events per predictor), a logistic regression tested whether psychometric dimensions were associated with hospitalization. The model was significant, χ^2^(8) = 19.35, *p* = 0.013 (Nagelkerke R^2^ = 0.192). Higher DERS scores were associated with increased odds of hospitalization, OR = 1.03, *p* = 0.021, while educational level was inversely associated, OR = 0.35, *p* = 0.004 (Table S30). Given the limited events-per-variable ratio, the model was re-estimated using Firth’s penalized-likelihood logistic regression, and the estimates were materially unchanged: higher DERS scores remained associated with greater odds of hospitalization, OR = 1.02, 95% CI [1.00, 1.05], *p* = 0.025, and higher educational level with lower odds, OR = 0.36, 95% CI [0.17, 0.73], *p* = 0.004; no other variable was significant (Table S38).

When ADHD severity and presentation were entered simultaneously as predictors of hospitalization (Table S31), the overall model was significant, χ^2^(5) = 20.88, *p* = 0.001 (Nagelkerke R^2^ = 0.163). Neither ADHD severity (OR = 1.71, *p* = 0.139) nor ADHD presentation (OR = 3.52, *p* = 0.104) was statistically significant when entered together. Educational level was the only significant independent correlate, OR = 0.38, *p* = 0.003.

**Figure 1:**
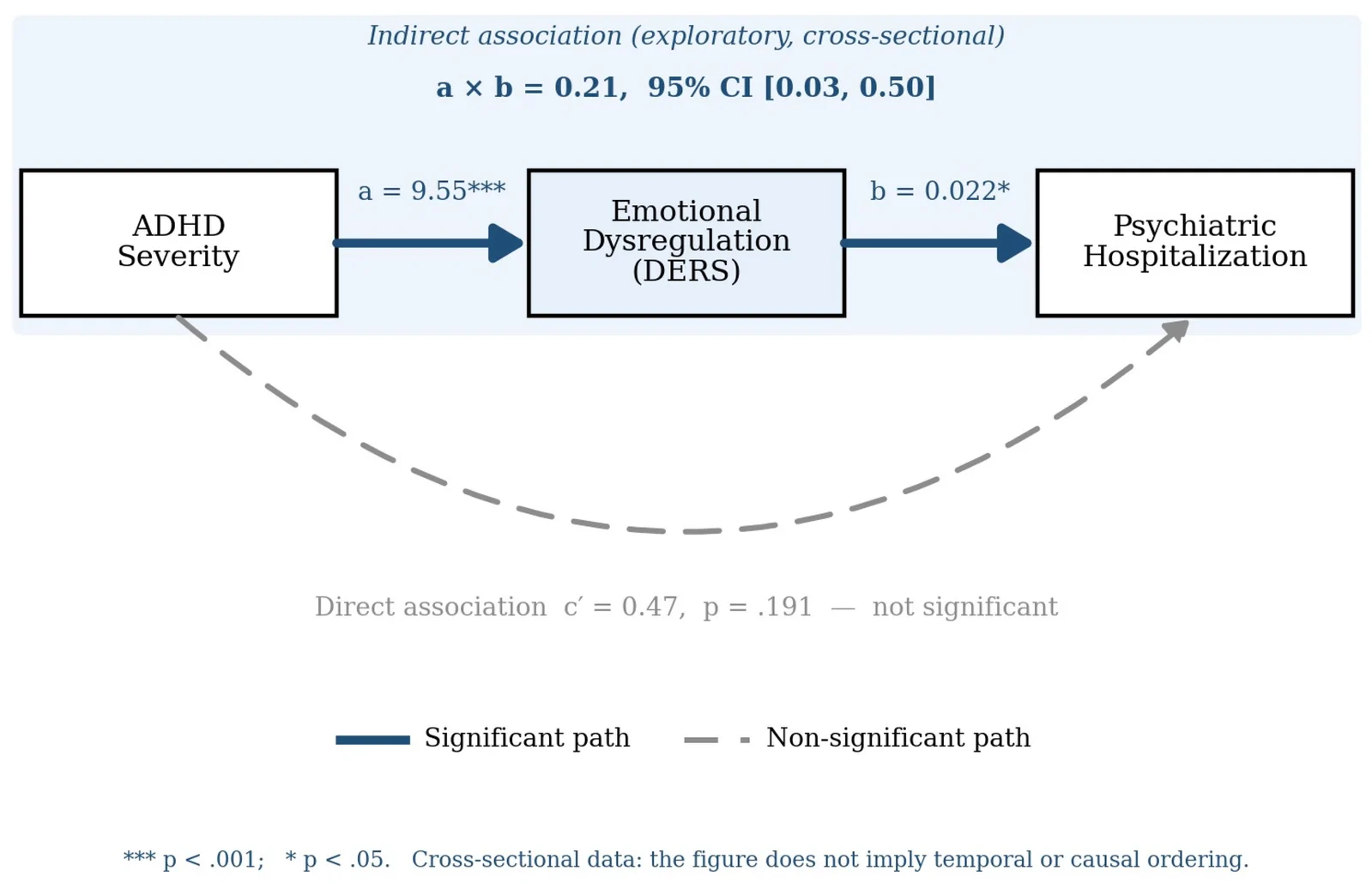
Exploratory cross-sectional indirect-effect model: ADHD Severity → Emotional Dysregulation (DERS) → Hospitalization. The a path (ADHD Severity → DERS: B = 9.55, *p* < 0.001) and b path (DERS → Hospitalization: B = 0.022, *p* = 0.025) were both significant. The direct effect (c′ path) was not significant (B = 0.47, *p* = 0.191). The indirect association was statistically significant (B = 0.21, 95% CI [0.03, 0.50]); given the cross-sectional design, it does not demonstrate mediation. Solid arrows denote statistically significant paths; the dashed arrow denotes the non-significant direct effect (c′). The intervening variable (DERS) and the significant a–b paths are highlighted.

An exploratory cross-sectional indirect-effect analysis (PROCESS Model 4; 5000 bootstrap samples) estimated whether emotional dysregulation statistically accounted for part of the association between ADHD severity and hospitalization, controlling for age, sex, and educational level (Table 8; Figure 1). ADHD severity was positively associated with emotional dysregulation (a path: B = 9.55, SE = 2.68, t = 3.56, *p* < 0.001). In turn, higher DERS scores were significantly associated with greater odds of hospitalization (b path: B = 0.022, SE = 0.010, z = 2.25, *p* = 0.025). The direct association between ADHD severity and hospitalization was not statistically significant after accounting for emotional dysregulation (c′ path: B = 0.47, SE = 0.36, *p* = 0.191). The indirect association between ADHD severity and hospitalization via emotional dysregulation was statistically significant (B = 0.21, BootSE = 0.12, 95% CI [0.03, 0.50]), as the confidence interval did not include zero. Given the cross-sectional design and the uncertain temporal ordering of the variables, this result indicates a statistical indirect association only and does not demonstrate mediation.

### 3.8. Duration of Untreated Illness by ADHD Presentation

An ANCOVA examined whether ADHD presentation was associated with DUI. ADHD presentation was not significantly associated with DUI, F(1, 90) = 1.17, *p* = 0.282, partial η^2^ = 0.013 (Table S32). Age emerged as a strong positive predictor, F(1, 90) = 144.00, *p* < 0.001, partial η^2^ = 0.615, whereas sex and educational level were not significant.

## 4. Discussion

The present study investigated psychometric characteristics, psychiatric comorbidities, and clinical correlates of ADHD presentations and symptom severity, using routinely collected clinical data from an adult sample in Milan, Italy. The findings, obtained in a specialist outpatient sample, are consistent with the heterogeneous nature of adult ADHD and with the value of integrating categorical and dimensional approaches when evaluating clinical complexity; given the cross-sectional design, all findings describe associations rather than causal or temporal relationships.

### 4.1. Differences Between Presentations in Emotional and Depressive Profiles

The multivariate comparison of psychometric profiles between ADHD presentations did not reach statistical significance. In exploratory univariate analyses, the Combined presentation showed modestly higher emotional dysregulation (DERS) than the Inattentive presentation, whereas the difference in depressive symptoms (ZUNG) was not significant. The difference in emotional dysregulation was small (partial η^2^ = 0.029) and did not survive correction for multiple testing; it therefore indicates, at most, a modest mean difference in a specific dimension rather than a broadly greater overall clinical burden of the Combined presentation, although its direction is consistent with previous studies [11,14]. The direction of the difference in emotional regulation is also coherent with factor-analytic evidence identifying emotional dysregulation as a dimension closely linked to hyperactive-impulsive symptomatology [17], as well as cluster-analytic studies showing that elevated emotional dysregulation is disproportionately represented within the Combined presentation [16].

The absence of significant differences between presentations in anxiety and problematic internet use should not be interpreted as evidence that these domains are determined by other factors, such as overall severity or comorbidity, as this was not directly tested; it may also reflect limited statistical power given the relatively small Inattentive group.

### 4.2. Substance Use Disorder and the Combined Presentation

A clinically relevant finding, and the most robust result of the comparison between presentations (it remained significant after Holm correction), was the markedly increased likelihood of substance use disorder in the Combined presentation, with nearly fivefold greater odds of SUD than the Inattentive presentation (30.4% vs. 8.3%). The wide confidence interval (OR 95% CI 1.58–14.49), however, indicates that the magnitude of this association is estimated imprecisely. This is consistent with evidence linking Combined ADHD to increased rates of substance misuse [12,48] and is compatible with neurobiological models proposing that impulsivity, reward-processing abnormalities, and deficient inhibitory control increase vulnerability to addictive behaviors [6]. Emotional dysregulation may further contribute by reinforcing maladaptive affect-based coping strategies, although this hypothesis was not directly tested here. The co-occurrence of ADHD and SUD is also clinically important beyond its prevalence: recent Italian real-world evidence indicates that comorbid alcohol and cannabis use disorders are associated with reduced clinical response to treatment in adults with ADHD [49], underscoring the contribution of SUD to the broader clinical complexity of adult ADHD and the need for its systematic assessment in specialist services [5]. Higher educational attainment was associated with lower odds of SUD; however, as discussed in Section 4.6, education may itself be influenced by ADHD severity and developmental history, so this association should not be interpreted as a protective effect.

### 4.3. ADHD Severity and Its Clinical Correlates

Clinician-rated ADHD severity was associated with several dimensional and categorical outcomes. The most robust associations—those surviving Holm correction—were with emotional dysregulation and SUD; associations with depressive symptoms, problematic internet use, and quality of life survived FDR control only, and the association with trait anxiety did not survive correction. Sensitivity analyses with dummy-coded severity were consistent with a graded increase in emotional dysregulation and SUD across severity levels, although the clearest contrast was between severe and mild ADHD, and the moderate-versus-mild differences were not individually significant. These cross-sectional findings are consistent with dimensional models of ADHD, in which symptom burden conveys clinically relevant information beyond categorical presentation [50], but they do not establish that severity has prognostic value, which would require prospective data. By contrast, sleep disturbance was not significantly associated with ADHD severity, although clinically significant insomnia remains a frequent and clinically relevant complaint in real-world psychiatric outpatient populations [51].

The positive association between ADHD severity and problematic internet use is consistent with longitudinal evidence linking greater ADHD symptom burden to the subsequent development of internet addiction [21,22]. A possible, untested explanation is that impulsivity and altered reward sensitivity facilitate excessive engagement in immediately reinforcing online activities [23,24].

Greater ADHD severity was unexpectedly associated with lower odds of a formal anxiety disorder diagnosis. This finding did not survive correction for multiple testing (Holm-adjusted *p* = 0.127) and should be regarded as exploratory and hypothesis-generating; the available data do not allow a definitive interpretation, and several alternative explanations, none of which could be directly tested here, should be considered. First, diagnostic overshadowing: in patients with more severe and conspicuous ADHD symptoms, anxiety-related features that overlap with ADHD (e.g., restlessness, concentration difficulties, and sleep problems) may be attributed to ADHD itself rather than coded as a separate disorder. Although diagnoses referred to the lifetime period, they were established retrospectively at a single interview, so anxiety disorders that were present earlier, or that fluctuated over time, may have been under-reported or overshadowed by current ADHD symptoms. Second, referral bias: patients with milder ADHD may be more likely to reach a specialist ADHD service when prominent anxiety contributes to their impairment, whereas patients with severe ADHD may be referred on the basis of ADHD symptoms alone. Third, under-recognition or under-reporting of anxiety symptoms by patients with more severe ADHD. Fourth, measurement factors: notably, severity was positively (though not robustly) associated with dimensional trait anxiety (STAI-T), suggesting that the inverse association may concern categorical diagnostic attribution rather than anxiety symptom levels. Finally, constructs such as emotional numbing, alexithymia, or impaired emotional awareness have been proposed, but they were not assessed and remain speculative. This observation therefore requires replication in independent cohorts using standardized lifetime diagnostic assessment.

### 4.4. Emotional Dysregulation and Psychiatric Hospitalization: An Exploratory Cross-Sectional Indirect Association

In an exploratory analysis, a statistically significant cross-sectional indirect association was observed between ADHD severity and psychiatric hospitalization via emotional dysregulation, whereas the direct association was not significant. This result must be interpreted with considerable caution. Cross-sectional analyses of mediation can yield substantially biased estimates of longitudinal mediation parameters and may suggest indirect effects even when none exist over time [46,47]. Moreover, hospitalization was recorded as a lifetime event whose timing could not be established and which may therefore have preceded the assessment of both ADHD severity and emotional dysregulation; in that case, the hypothesized sequence (severity → emotional dysregulation → hospitalization) would be temporally implausible, and reverse or bidirectional associations—for instance, the experience of hospitalization or the severity of the underlying episode influencing self-reported emotional dysregulation—cannot be excluded. The analysis therefore does not test mediation and should be regarded only as describing a pattern of cross-sectional associations. Finally, psychiatric hospitalization is a complex outcome influenced by many clinical and environmental factors not included in the model (e.g., bipolar or psychotic comorbidity, suicidality, personality disorders, social support, and service availability), so residual confounding is likely.

With these caveats, the findings are consistent with the view that emotional dysregulation is a clinically relevant dimension in adults with ADHD [5,18], and they support including a systematic assessment of emotional dysregulation in the evaluation of adults with ADHD. Whether interventions targeting emotional dysregulation—such as dialectical behavior therapy, emotion regulation-focused cognitive behavioral approaches, or pharmacological treatment—could reduce the risk of hospitalization could not be addressed here, since no treatment was evaluated and treatment response was not analyzed; this hypothesis would require prospective and interventional studies. Likewise, the inverse association between educational level and hospitalization should be interpreted as a cross-sectional correlate rather than a protective effect.

### 4.5. Sex Effects

Female sex was independently associated with greater depressive symptom severity and poorer quality of life, after adjustment for ADHD presentation or severity. These observations are consistent with literature describing a female ADHD phenotype with a predominance of internalizing symptoms, higher rates of depression and anxiety, and greater functional impairment [16,20]. This profile may contribute to delayed or missed diagnosis, as mood and anxiety symptoms frequently overshadow the underlying neurodevelopmental disorder [18]. Clinicians should maintain a high index of suspicion for ADHD in women with persistent or treatment-resistant affective symptoms.

These differences may also be partly shaped by gender-related help-seeking and diagnostic pathways. Women are more likely than men to seek help for emotional and internalizing difficulties, and ADHD in girls and women tends to be recognized later, frequently after an initial diagnosis—and treatment—of depression or anxiety [52,53]. Consequently, women may reach a specialist ADHD service after longer periods of unrecognized ADHD, with the cumulative burden of compensatory efforts (including camouflaging of symptoms) and of secondary affective difficulties, which may contribute to greater depressive symptoms and lower quality of life at the time of assessment. At the same time, women referred to a specialist service may represent a selected subgroup with more prominent internalizing symptoms, and sex differences in self-report response styles may also contribute. As age at diagnosis and DUI were not compared by sex in the present sample, these interpretations remain hypothetical.

The association between female sex and increased reporting of family ADHD history may reflect greater awareness of ADHD within families or sex-related differences in healthcare-seeking behavior, although true sex-related differences in familial aggregation cannot be excluded.

### 4.6. Educational Level

Educational attainment was one of the most consistent correlates across analyses. Higher educational level was associated with lower odds of substance use disorder, psychiatric hospitalization, and current pharmacological treatment and, at trend level, of sleep disturbance, but with higher odds of an anxiety disorder diagnosis, potentially reflecting greater awareness, recognition, and reporting of internal emotional experiences [12].

Several non-mutually exclusive explanations may account for these associations. First, cognitive reserve: higher educational attainment may both reflect and further build cognitive and executive resources, as well as learned organizational strategies, that help compensate for ADHD-related deficits and buffer their functional consequences. Second, social support and socioeconomic resources: more educated individuals are more likely to have stable employment, supportive social networks, and material resources, which may reduce exposure to stressors that precipitate substance misuse or psychiatric crises. Third, healthcare access and health literacy: higher education is associated with earlier help-seeking and better access to specialist care and psychotherapy, as well as with a greater ability to recognize and verbalize internal states—which may also explain the higher odds of anxiety diagnoses and, possibly through preferences for non-pharmacological approaches, the lower odds of current pharmacological treatment. At the same time, educational attainment cannot be regarded simply as a protective exposure: it is itself influenced by ADHD severity, early-onset comorbidity (including early substance use), and developmental history [7], so that reverse causation and confounding are likely. Notably, education was not associated with emotional dysregulation, whereas its inverse association with SUD was robust to the coding of educational level, being evident for both high-school and university education relative to low education. Whether educational and vocational support modifies clinical outcomes in adults with ADHD remains to be tested prospectively.

### 4.7. Duration of Untreated Illness

Duration of untreated illness was primarily explained by age, supporting evidence that many adults experience substantial delays before an ADHD diagnosis, particularly those who reached adulthood before ADHD became widely recognized in adult psychiatric practice [54,55]. The absence of associations with presentation or severity is compatible with the hypothesis that diagnostic delay is driven largely by historical and healthcare-system factors rather than by clinical phenotype. However, DUI was available for fewer than half of the sample (*n* = 97) and is structurally dependent on current age; these findings should therefore be interpreted with caution.

### 4.8. Limitations

Several limitations should be considered. First, the retrospective cross-sectional design does not permit assessment of temporal relationships or causal pathways; all findings should be interpreted as associations. In particular, the exposure, intervening variable, and outcome of the indirect-effect model were not measured in a temporally ordered way, and hospitalization was a historical rather than a prospectively assessed outcome; longitudinal studies with prospective follow-up are required to determine whether emotional dysregulation is associated with future hospitalization or clinical deterioration.

Second, the sample was recruited from a single specialist outpatient ADHD service in Milan and is subject to referral bias. Adults referred to specialist services may have greater symptom burden, more psychiatric comorbidity, and greater functional impairment than community-dwelling adults with ADHD, and the study did not include undiagnosed adults, community samples, or individuals with milder presentations. The findings therefore cannot be generalized to community-dwelling adults with ADHD or to adults managed in primary care, general psychiatric settings, or other healthcare systems.

Third, the distribution of ADHD presentations was highly unbalanced. The Predominantly Hyperactive-Impulsive presentation (*n* = 3) was excluded from all comparisons between presentations, and no conclusions should be extended to this presentation; moreover, the relatively small Inattentive group (*n* = 48) limited statistical power. The study therefore does not provide a comprehensive evaluation of all DSM-5 presentations.

Fourth, ADHD severity was based on a single clinician-rated ordinal DSM-5 specifier, whose inter-rater reliability was not assessed, and it was modelled as a linear predictor; sensitivity analyses with dummy-coded severity supported a graded pattern but indicated that the clearest contrast was between severe and mild ADHD, and measurement error in this central variable may have attenuated or distorted the observed associations.

Fifth, a large number of statistical models were estimated. Although multiplicity was addressed within predefined families of outcomes, only a minority of associations survived Holm correction; all other results, and all secondary analyses, should be regarded as exploratory and require confirmation in independent samples.

Sixth, the study relied on routinely collected clinical information rather than data specifically collected for research purposes, with non-negligible missing data for some variables (e.g., DUI) and possible variability in documentation and assessment procedures over time. Complete-case analysis may have introduced selection bias if data were not missing completely at random; although participants with complete and incomplete psychometric data were comparable in age, education, ADHD presentation, and severity, women were over-represented among those with complete data (56.5% vs. 33.1%), which may limit the generalizability of the psychometric findings despite adjustment for sex in all models. In addition, the multivariable hospitalization model was based on a small number of events (24 events; 3.0 events per predictor); although Firth penalized estimates were materially unchanged, these results should be considered exploratory.

Seventh, several outcomes (depression, anxiety, emotional dysregulation, internet use, impulsivity, and quality of life) were assessed with self-report questionnaires, which are subject to response bias (e.g., social desirability and symptom over- or under-reporting) and, for retrospective information such as symptom onset, recall bias; no objective or clinician-rated measures of these dimensions were available, and questionnaire administration was not governed by research-grade quality-control procedures. In addition, some participants may already have been receiving psychiatric treatment when the questionnaires were completed, which may have influenced their scores.

Finally, although comorbid psychiatric diagnoses referred to the lifetime period, they were assessed retrospectively at a single interview and may underestimate lifetime diagnostic burden; together with possible diagnostic overshadowing, this may have led to under-recognition of some comorbidities, particularly anxiety disorders. Other relevant confounders of psychiatric hospitalization (e.g., bipolar or psychotic disorders, suicidality, personality disorders, and social support) were not included in the models. In addition, ASD diagnoses were obtained through heterogeneous pathways (external private centres or structured assessment within our service), which may have introduced variability in diagnostic ascertainment.

## 5. Conclusions

In this single-centre, cross-sectional study of adults attending a specialist Italian ADHD service, the Combined presentation was associated with substantially higher odds of substance use disorder than the Inattentive presentation, whereas psychometric profiles did not differ significantly between presentations at the multivariate level, and the higher emotional dysregulation in the Combined presentation was small and not robust to correction for multiple testing. Greater clinician-rated ADHD severity was associated with greater emotional dysregulation and higher odds of substance use disorder. In an exploratory analysis, emotional dysregulation was involved in a cross-sectional indirect association between ADHD severity and psychiatric hospitalization; this pattern does not establish mediation and requires testing in prospective studies with temporally ordered measurements. Overall, the findings support a systematic assessment of ADHD presentation, severity, emotional dysregulation, and substance use in adults with ADHD, and provide hypotheses for longitudinal, multicentre research.

## Figures and Tables

**Table 1:** Socio-Demographic and Clinical Characteristics of the Sample, Overall and by ADHD Presentation.

Characteristic	Total Sample (*N* = 219)	Combined (*n* = 168)	Inattentive (*n* = 48)	Available Data, *n* (Missing, *n*)
Age, years, M (SD)	26.16 (4.89)	26.20 (4.92)	26.15 (4.93)	219 (0)
Female sex, *n* (%)	92 (42.2)	72 (43.1)	18 (37.5)	218 (1)
**Educational level, *n* (%)**				219 (0)
Low education	34 (15.5)	31 (18.5)	3 (6.2)	
High school	120 (54.8)	87 (51.8)	31 (64.6)	
University degree	65 (29.7)	50 (29.8)	14 (29.2)	
ADHD presentation, *n* (%)	C: 168 (76.7); I: 48 (21.9); HI: 3 (1.4)	—	—	219 (0)
**ADHD severity (DIVA-5), *n* (%)**				219 (0)
Mild	28 (12.8)	11 (6.5)	17 (35.4)	
Moderate	133 (60.7)	103 (61.3)	27 (56.2)	
Severe	58 (26.5)	54 (32.1)	4 (8.3)	
**Psychiatric comorbidity, *n* (%)**				
Mood disorder	40 (18.3)	28 (16.7)	11 (22.9)	219 (0)
Bipolar disorder	6 (2.7)	5 (3.0)	1 (2.1)	219 (0)
Anxiety disorder	64 (29.2)	47 (28.0)	16 (33.3)	219 (0)
Substance use disorder (current)	57 (26.0)	51 (30.4)	4 (8.3)	219 (0)
Stimulants	36 (63.2)	31 (60.8)	3 (75.0)	57
Novel psychoactive substances	13 (22.8)	13 (25.5)	0	
Ecstasy	7 (12.3)	7 (13.7)	0	
Opioids	3 (5.3)	3 (5.9)	0	
Cannabinoids	1 (1.8)	0	1 (25.0)	
Autism spectrum disorder	18 (8.2)	17 (10.1)	1 (2.1)	219 (0)
Other psychiatric disorders	98 (44.7)	77 (45.8)	19 (39.6)	219 (0)
Sleep disturbance	161 (73.9)	130 (77.4)	29 (61.7)	218 (1)
**Symptom screening, *n* (%)**				
Depressive symptoms, ZUNG total ≥ 40	146 (76.0)	118 (78.7)	27 (69.2)	192 (27)
Positive MDQ screening (bipolar spectrum)	51 (26.4)	46 (30.9)	4 (9.8)	193 (26)
Anxiety symptoms, STAI-State ≥ 40	173 (89.2)	135 (88.8)	36 (92.3)	194 (25)
Anxiety symptoms, STAI-Trait ≥ 40	185 (95.4)	146 (96.1)	36 (92.3)	194 (25)
Psychiatric hospitalization (lifetime), *n* (%)	32 (14.6)	30 (17.9)	2 (4.2)	219 (0)
Current pharmacological treatment, *n* (%)	136 (62.1)	111 (66.1)	25 (52.1)	219 (0)
Current psychotherapy, *n* (%)	94 (42.9)	75 (44.6)	18 (37.5)	219 (0)
**Psychometric measures, M (SD)**				
DERS total	114.70 (24.05)	117.39 (24.53)	104.50 (19.73)	208 (11)
ZUNG total score	47.46 (10.95)	48.49 (11.13)	44.26 (9.02)	192 (27)
STAI-State	54.49 (12.46)	54.72 (12.99)	54.08 (10.26)	194 (25)
STAI-Trait	58.72 (10.25)	58.89 (10.29)	58.31 (10.41)	194 (25)
IAT total	53.27 (15.44)	53.98 (16.06)	50.39 (13.19)	201 (18)
AAQoL total	43.53 (15.22)	43.12 (15.75)	44.78 (11.46)	198 (21)
BIS-11 total	82.30 (16.69)	83.96 (15.80)	76.59 (19.01)	204 (15)
DUI, years, M (SD)	12.26 (6.62)	12.16 (6.57)	13.11 (6.72)	97 (122)

Note. C = Combined; I = Predominantly Inattentive; HI = Predominantly Hyperactive-Impulsive. Available *n* refers to participants with non-missing data; regression models may include slightly fewer participants because of missing covariates. Substance categories refer to the substance of problematic use or dependence among participants with current SUD (percentages of those with SUD; categories not mutually exclusive). Cut-offs: ZUNG raw total score ≥ 40 (SDS index ≥ 50); STAI ≥ 40. Psychiatric comorbidities are not mutually exclusive; “other psychiatric disorders” includes all diagnoses other than mood, bipolar, anxiety, and autism spectrum disorders. MDQ = Mood Disorder Questionnaire; STAI = State-Trait Anxiety Inventory. SUD = substance use disorder; DUI = duration of untreated illness.

**Table 3:** Multivariate Effects of ADHD Presentation on Psychometric Variables (MANCOVA).

Effect	Wilks’ Λ	F	df1	df2	*p*	Partial η^2^
ADHD Presentation	0.939	2.10	5	162	0.068	0.061
Age	0.976	0.79	5	162	0.558	0.024
Sex	0.929	2.47	5	162	0.034	0.071
Education	0.976	0.81	5	162	0.544	0.024

Note. Dependent variables included ZUNG (raw total score), STAI-State, STAI-Trait, DERS total, and IAT total. Age, sex, and education were entered as covariates. Partial η^2^ values represent effect sizes.

**Table 4:** Univariate Effects of ADHD Presentation on Psychometric Measures.

Outcome	F	df	*p*	Partial η^2^
ZUNG	3.09	1, 166	0.081	0.018
STAI-State	0.04	1, 166	0.853	0.000
STAI-Trait	0.03	1, 166	0.856	0.000
DERS	4.93	1, 166	0.028	0.029
IAT Total	1.09	1, 166	0.299	0.006

Note. ZUNG = raw total score. Values reflect Type III sums of squares. Analyses adjusted for age, sex, and education.

**Table 5:** Logistic Regression of Substance Use Disorder on ADHD Presentation and Covariates.

Predictor	B	SE	Wald	*p*	OR	95% CI for OR
ADHD Presentation (1 = Combined)	1.566	0.565	7.68	0.006	4.79	[1.58, 14.49]
Age	0.016	0.036	0.20	0.655	1.02	[0.95, 1.09]
Sex (1 = Female)	−0.627	0.363	2.99	0.084	0.53	[0.26, 1.09]
Education	−1.033	0.278	13.83	<0.001	0.36	[0.21, 0.61]

Note. *N* = 215.

**Table 6:** Summary of Linear Regression Models Examining Associations Between ADHD Severity and Dimensional Outcomes.

Outcome	B	SE	β	t	*p*	R^2^
Emotional Dysregulation	9.546	2.683	0.248	3.56	<0.001	0.066
Depressive Symptoms	2.955	1.241	0.171	2.38	0.018	0.091
Internet Addiction	4.545	1.842	0.176	2.47	0.014	0.053
State Anxiety	2.440	1.454	0.124	1.68	0.095	0.039
Trait Anxiety	2.386	1.190	0.147	2.01	0.046	0.048
Duration of Untreated Illness	0.082	0.718	0.007	0.11	0.910	0.622 *

Note. All models adjusted for age, sex, and educational level. * In the Duration of Untreated Illness model, variance was primarily explained by age (β = 0.798, *p* < 0.001).

**Table 7:** Summary of Logistic Regression Models Examining Associations Between ADHD Severity and Psychiatric Comorbidities.

Outcome	B	SE	Wald	*p*	OR	95% CI for OR	Nagelkerke R^2^
Substance Use Disorder	0.933	0.296	9.95	0.002	2.54	[1.42, 4.54]	0.202
Anxiety Disorder	−0.594	0.265	5.00	0.025	0.55	[0.33, 0.93]	0.115
Mood Disorder	0.599	0.326	3.39	0.066	1.82	[0.96, 3.45]	0.033
Sleep Disorder	0.315	0.264	1.42	0.233	1.37	[0.82, 2.30]	0.053
Other Psychiatric Disorders	0.644	0.411	2.45	0.118	1.90	[0.85, 4.26]	0.084
Autism Spectrum Disorder	−0.433	0.409	1.12	0.290	0.65	[0.29, 1.45]	0.062

Note. All models adjusted for age, sex, and educational level. OR = Odds Ratio. R^2^ values refer to Nagelkerke R^2^.

**Table 8:** Exploratory Cross-Sectional Indirect-Effect Model: ADHD Severity → Emotional Dysregulation → Hospitalization.

Path	B	SE	z/t	*p*	95% CI
*a* path: Severity → DERS	9.55	2.68	3.56	<0.001	[4.26, 14.84]
*b* path: DERS → Hospitalization	0.022	0.010	2.25	0.025	[0.003, 0.042]
*c*′ path: Severity → Hospitalization (direct)	0.473	0.362	1.31	0.191	[−0.236, 1.181]
Indirect effect (bootstrapped)	0.211	0.121	—	—	[0.032, 0.501]

Note. *N* = 208. Bootstrap samples = 5000. Because all variables were assessed cross-sectionally and hospitalization may have preceded the assessment, the indirect effect represents a statistical association only and should not be interpreted as evidence of mediation.

## Data Availability

The data presented in this study are available on request from the corresponding author due to privacy restrictions.

## Supplementary Materials

The following supporting information can be downloaded at: https://www.mdpi.com/article/10.3390/jcm15207953/s1, Tables S1–S38, including the completed STROBE checklist (Table S35).

## Abbreviations

The following abbreviations are used in this manuscript:

AAQoL	Adult ADHD Quality of Life Questionnaire
ADHD	Attention-Deficit/Hyperactivity Disorder
ASD	Autism Spectrum Disorder
ASST	Azienda Socio Sanitaria Territoriale
BIS-11	Barratt Impulsiveness Scale-11
CAT-Q	Camouflaging Autistic Traits Questionnaire
CBT	Cognitive-Behavioral Therapy
CI	Confidence Interval
DERS	Difficulties in Emotion Regulation Scale
DIVA	Diagnostic Interview for ADHD in Adults
DSM-5	Diagnostic and Statistical Manual of Mental Disorders, Fifth Edition
DUI	Duration of Untreated Illness
IAT	Internet Addiction Test
OR	Odds Ratio
SD	Standard Deviation
SPSS	Statistical Package for the Social Sciences
STAI	State-Trait Anxiety Inventory
STAI-Y1	State Anxiety Inventory, Form Y1
STAI-Y2	Trait Anxiety Inventory, Form Y2
WURS	Wender Utah Rating Scale

## References

[1] Simon V., Czobor P., Bálint S., Mészáros Á., Bitter I. (2009). Prevalence and correlates of adult attention-deficit hyperactivity disorder: Meta-analysis. Br. J. Psychiatry.

[2] Fayyad J., Sampson N.A., Hwang I., Adamowski T., Aguilar-Gaxiola S., Al-Hamzawi A., Andrade L.H., Borges G., de Girolamo G., Florescu S. (2017). The descriptive epidemiology of DSM-IV adult ADHD in the World Health Organization World Mental Health Surveys. ADHD Atten. Deficit Hyperact. Disord..

[3] Song P., Zha M., Yang Q., Zhang Y., Li X., Rudan I. (2021). The prevalence of adult attention-deficit hyperactivity disorder: A global systematic review and meta-analysis. J. Glob. Health.

[4] Popit S., Serod K., Locatelli I., Stuhec M. (2024). Prevalence of attention-deficit hyperactivity disorder (ADHD): Systematic review and meta-analysis. Eur. Psychiatry.

[5] Cortese S., Bellgrove M.A., Brikell I., Franke B., Goodman D.W., Hartman C.A., Larsson H., Levin F.R., Ostinelli E.G., Parlatini V. (2025). Attention-deficit/hyperactivity disorder (ADHD) in adults: Evidence base, uncertainties and controversies. World Psychiatry.

[6] Barkley R.A., Murphy K.R., Fischer M. (2008). ADHD in Adults: What the Science Says.

[7] Fredriksen M., Dahl A.A., Martinsen E.W., Klungsoyr O., Faraone S.V., Peleikis D.E. (2014). Childhood and persistent ADHD symptoms associated with educational failure and long-term occupational disability in adult ADHD. ADHD Atten. Deficit Hyperact. Disord..

[8] Halmøy A., Fasmer O.B., Gillberg C., Haavik J. (2009). Occupational outcome in adult ADHD: Impact of symptom profile, comorbid psychiatric problems, and treatment—A cross-sectional study of 414 clinically diagnosed adult ADHD patients. J. Atten. Disord..

[9] American Psychiatric Association (2013). Diagnostic and Statistical Manual of Mental Disorders.

[10] Millstein R.B., Wilens T.E., Biederman J., Spencer T.J. (1997). Presenting ADHD symptoms and subtypes in clinically referred adults with ADHD. J. Atten. Disord..

[11] Søndergaard H.M., Thomsen P.H., Pedersen E.M., Bentz M., Poulsen A.E., Kragballe M., Hougaard E. (2016). Associations of age, sex, and subtypes with ADHD symptoms and related comorbidity in a Danish sample of clinically referred adults. J. Atten. Disord..

[12] Piñeiro-Dieguez B., Balanzá-Martínez V., García-García P., Soler-López B. (2016). Psychiatric comorbidity at the time of diagnosis in adults with ADHD: The CAT study. J. Atten. Disord..

[13] Kessler R.C., Adler L., Barkley R., Biederman J., Conners C.K., Demler O., Faraone S.V., Greenhill L.L., Howes M.J., Secnik K. (2006). The prevalence and correlates of adult ADHD in the United States: Results from the National Comorbidity Survey Replication. Am. J. Psychiatry.

[14] Tsai F.-J., Tseng W.-L., Yang L.-K., Gau S.S.-F., Hwang H.-F. (2019). Psychiatric comorbid patterns in adults with attention-deficit hyperactivity disorder: Treatment effect and subtypes. PLoS ONE.

[15] Mirza H., Al-Huseini S., Al-Jamoodi S., Al-Haddabi R., Al-Mawali A., Al-Adawi S. (2021). The socio-demographic and clinical profiles of adult ADHD patients in a university hospital in Oman. Sultan Qaboos Univ. Med. J..

[16] Hirsch O., Chavanon M.-L., Christiansen H. (2019). Emotional dysregulation subgroups in patients with adult attention-deficit/hyperactivity disorder (ADHD): A cluster analytic approach. Sci. Rep..

[17] Adler L.A., Faraone S.V., Spencer T.J., Berglund P., Alperin S., Kessler R.C. (2017). The structure of adult ADHD. Int. J. Methods Psychiatr. Res..

[18] Reif A. (2025). The emotional side of adult ADHD. World Psychiatry.

[19] Huang J., Mauche N., Ahlers E., Richter M., Cao H., Voss M., Hegerl U. (2024). The impact of emotional dysregulation and comorbid depressive symptoms on clinical features, brain arousal, and treatment response in adults with ADHD. Front. Psychiatry.

[20] Pallucchini A., Carli M., Scarselli M., Weiss F., Maremmani I. (2021). Symptomatological variants and related clinical features in adult attention deficit hyperactive disorder. Int. J. Environ. Res. Public Health.

[21] Wang B., Yao N., Zhou X., Liu J., Lv Z. (2017). The association between attention deficit/hyperactivity disorder and internet addiction: A systematic review and meta-analysis. BMC Psychiatry.

[22] Zhou B., Zhang W., Li W., Nie J., Xiao L. (2020). Motivational but not executive dysfunction in attention deficit/hyperactivity disorder predicts internet addiction: Evidence from a longitudinal study. Psychiatry Res..

[23] Ishii S., Takagi S., Kobayashi N., Mimura M. (2023). Hyperfocus symptom and internet addiction in individuals with attention-deficit/hyperactivity disorder trait. Front. Psychiatry.

[24] Li W., Zhang W., Xiao L., Nie J. (2016). The association of internet addiction symptoms with impulsiveness, loneliness, novelty seeking and behavioral inhibition system among adults with attention-deficit/hyperactivity disorder (ADHD). Psychiatry Res..

[25] Chen C.-Y., Lee K.Y., Fung X. (2024). Problematic use of internet associates with poor quality of life via psychological distress in individuals with ADHD. Psychol. Res. Behav. Manag..

[26] Silva M.A., Chaim T.M., Cavalett M., Bressan R.A., Lacerda A.L.T. (2014). Health-related quality of life in adults with attention-deficit and hyperactivity disorder. Med. Express.

[27] Murray A.J. (2025). Suspected and diagnosed ADHD quality of life differences: An investigative study of New Zealand adults. Discov. Psychol..

[28] Pan M.-R., Zhang S.-Y., Chen C.-L., Qiu S.-W., Liu L., Li H.-M., Zhao M.-J., Dong M., Si F.-F., Wang Y.-F. (2023). Bidirectional associations between maladaptive cognitions and emotional symptoms, and their mediating role on the quality of life in adults with ADHD: A mediation model. Front. Psychiatry.

[29] Gjervan B., Torgersen T., Rasmussen K. (2014). ADHD symptoms are differentially related to specific aspects of quality of life. J. Atten. Disord..

[30] von Elm E., Altman D.G., Egger M., Pocock S.J., Gøtzsche P.C., Vandenbroucke J.P. (2007). The Strengthening the Reporting of Observational Studies in Epidemiology (STROBE) statement: Guidelines for reporting observational studies. Lancet.

[31] Kooij J.J.S., Francken M.H., Bron T.I. (2019). Diagnostic Interview for ADHD in Adults (DIVA-5).

[32] Ward M.F., Wender P.H., Reimherr F.W. (1993). The Wender Utah Rating Scale: An aid in the retrospective diagnosis of childhood attention deficit hyperactivity disorder. Am. J. Psychiatry.

[33] Zung W.W.K. (1965). A self-rating depression scale. Arch. Gen. Psychiatry.

[34] Spielberger C.D., Gorsuch R.L., Lushene R., Vagg P.R., Jacobs G.A. (1983). Manual for the State-Trait Anxiety Inventory.

[35] Gratz K.L., Roemer L. (2004). Multidimensional assessment of emotion regulation and dysregulation: Development, factor structure, and initial validation of the Difficulties in Emotion Regulation Scale. J. Psychopathol. Behav. Assess..

[36] Young K.S. (1998). Internet addiction: The emergence of a new clinical disorder. Cyberpsychol. Behav..

[37] Brod M., Johnston J., Able S., Swindle R. (2006). Validation of the Adult Attention-Deficit/Hyperactivity Disorder Quality-of-Life Scale (AAQoL): A Disease-Specific Quality-of-Life Measure. Qual. Life Res..

[38] Patton J.H., Stanford M.S., Barratt E.S. (1995). Factor structure of the Barratt Impulsiveness Scale. J. Clin. Psychol..

[39] Hirschfeld R.M.A., Williams J.B.W., Spitzer R.L., Calabrese J.R., Flynn L., Keck P.E., Lewis L., McElroy S.L., Post R.M., Rapport D.J. (2000). Development and validation of a screening instrument for bipolar spectrum disorder: The Mood Disorder Questionnaire. Am. J. Psychiatry.

[40] First M.B., Williams J.B.W., Benjamin L.S., Spitzer R.L. (2016). Structured Clinical Interview for DSM-5 Personality Disorders (SCID-5-PD).

[41] Holm S. (1979). A simple sequentially rejective multiple test procedure. Scand. J. Stat..

[42] Benjamini Y., Hochberg Y. (1995). Controlling the false discovery rate: A practical and powerful approach to multiple testing. J. R. Stat. Soc. Ser. B.

[43] Firth D. (1993). Bias reduction of maximum likelihood estimates. Biometrika.

[44] Peduzzi P., Concato J., Kemper E., Holford T.R., Feinstein A.R. (1996). A simulation study of the number of events per variable in logistic regression analysis. J. Clin. Epidemiol..

[45] Hayes A.F. (2013). Introduction to Mediation, Moderation, and Conditional Process Analysis: A Regression-Based Approach.

[46] Maxwell S.E., Cole D.A. (2007). Bias in cross-sectional analyses of longitudinal mediation. Psychol. Methods.

[47] Maxwell S.E., Cole D.A., Mitchell M.A. (2011). Bias in cross-sectional analyses of longitudinal mediation: Partial and complete mediation under an autoregressive model. Multivar. Behav. Res..

[48] Liebrenz M., Gamma A., Ivanov I., Buadze A., Eich D. (2015). Adult attention-deficit/hyperactivity disorder: Associations between subtype and lifetime substance use—A clinical study. F1000Research.

[49] Carbone M.G., Tripodi B., Matarese I., Bellini A., Rizzato R., Tagliarini C., Della Rocca F., De Dominicis F., Maremmani I., Maremmani A.G.I. (2026). Factors associated with reduced clinical response in adult ADHD: The role of alcohol and cannabis use disorders and autism spectrum disorder. J. Clin. Med..

[50] Faraone S.V., Banaschewski T., Coghill D., Zheng Y., Biederman J., Bellgrove M.A., Newcorn J.H., Gignac M., Al Saud N.M., Manor I. (2021). The World Federation of ADHD International Consensus Statement: 208 evidence-based conclusions about the disorder. Neurosci. Biobehav. Rev..

[51] Martiadis V., Pessina E., Martini A., Marzolla M., Bergesio C., Barbaro F., Cavallo A., Raffone F., Cattaneo C.I. (2026). Insomnia severity in psychiatric outpatients: Real-world Insomnia Severity Index data from an Italian community mental health center. Brain Sci..

[52] Quinn P.O., Madhoo M. (2014). A review of attention-deficit/hyperactivity disorder in women and girls: Uncovering this hidden diagnosis. Prim. Care Companion CNS Disord..

[53] Young S., Adamo N., Ásgeirsdóttir B.B., Branney P., Beckett M., Colley W., Cubbin S., Deeley Q., Farrag E., Gudjonsson G. (2020). Females with ADHD: An expert consensus statement taking a lifespan approach providing guidance for the identification and treatment of attention-deficit/hyperactivity disorder in girls and women. BMC Psychiatry.

[54] Torgersen T., Gjervan B., Rasmussen K. (2006). ADHD in adults: A study of clinical characteristics, impairment and comorbidity. Nord. J. Psychiatry.

[55] Brown T.E., Romero B., Sarocco P. (2019). The patient perspective: Unmet treatment needs in adults with attention-deficit/hyperactivity disorder. Prim. Care Companion J. Clin. Psychiatry.

