# Supplementary material for: ADHD Presentations and Severity in Adults: Psychometric Profiles, Psychiatric Comorbidities, and Emotional Dysregulation in Relation to Psychiatric Hospitalization
Source: J Clin Med. 2026 Oct 14;15(20):7953. doi: 10.3390/jcm15207953 (PMC13705952; doi:10.3390/jcm15207953)
Supplement: Supplementary file 1 [file jcm-15-07953-s001.zip › jcm-4502058-supplementary.pdf]

Supplementary material for: ADHD Presentations and Severity in Adults: Psychometric Profiles, Psychiatric Comorbidities, and Emotional Dysregulation in Relation to Psychiatric Hospitalization

Supplementary Material

The following tables are provided as Supplementary Material and are cited in the main text as Table S1–Table S38.

Table S1. Adjusted Marginal Means by ADHD Presentation

Outcome	Combined M (SE)	Inattentive M (SE)
ZUNG (total score)	48.63 (.91)	44.99 (1.86)
STAI-State	53.99 (1.06)	54.44 (2.17)
STAI-Trait	58.40 (.87)	58.77 (1.79)
DERS	117.00 (2.07)	106.50 (4.24)
IAT Total	54.13 (1.34)	50.93 (2.76)

Note. Estimated marginal means adjusted for age (*M* = 26.12), sex (*M* = 0.44), and education (*M* = 3.14). ZUNG = raw total score (range 20–80); N = 171.

Table S2. Logistic Regression Predicting Mood Disorder Comorbidity From ADHD Presentation and Covariates

Predictor	B	SE	Wald	p	OR	95% CI for OR
ADHD Presentation (1 = Combined)	-0.290	0.433	0.45	.502	0.75	[0.32, 1.75]
Age	-0.004	0.041	0.01	.925	1.00	[0.92, 1.08]
Sex (1 = Female)	-0.120	0.392	0.09	.759	0.89	[0.41, 1.91]
Education	-0.191	0.303	0.40	.530	0.83	[0.46, 1.50]

Note. *N* = 215. OR = Odds Ratio. CI = Confidence Interval.

Table S3. Logistic Regression Predicting Anxiety Disorder Comorbidity From ADHD Presentation and Covariates

Predictor	B	SE	Wald	p	OR	95% CI for OR
ADHD Presentation (1 = Combined)	-0.207	0.361	0.33	.567	0.81	[0.40, 1.65]
Age	0.020	0.034	0.36	.551	1.02	[0.96, 1.09]
Sex (1 = Female)	-0.361	0.324	1.24	.265	0.70	[0.37, 1.32]
Education	0.721	0.269	7.19	.007	2.06	[1.21, 3.48]

Note. N = 215.

Table S4. Logistic Regression Predicting Other Psychiatric Comorbidity From ADHD Presentation and Covariates

Predictor	B	SE	Wald	p	OR	95% CI for OR
ADHD Presentation (1 = Combined)	1.010	0.770	1.72	.190	2.74	[0.61, 12.42]
Age	0.103	0.050	4.23	.040	1.11	[1.01, 1.22]
Sex (1 = Female)	0.596	0.483	1.52	.218	1.81	[0.70, 4.68]
Education	-0.491	0.350	1.97	.161	0.61	[0.31, 1.22]

Note. N = 215.

Table S5. Logistic Regression Predicting Autism Spectrum Comorbidity From ADHD Presentation and Covariates

Predictor	B	SE	Wald	p	OR	95% CI for OR
ADHD Presentation (1 = Combined)	1.615	1.046	2.38	.123	5.03	[0.65, 39.07]
Age	-0.024	0.055	0.20	.657	0.98	[0.88, 1.09]
Sex (1 = Female)	0.373	0.501	0.55	.457	1.45	[0.54, 3.87]
Education	-0.614	0.382	2.58	.108	0.54	[0.26, 1.14]

Note. N = 215.

Table S6. Multiple Linear Regression Predicting Emotional Dysregulation From ADHD Severity and Covariates

Predictor	B	SE	β	t	p	95% CI for B
ADHD Severity	9.546	2.683	.248	3.56	< .001	[4.26, 14.84]
Age	0.120	0.365	.024	0.33	.743	[-0.60, 0.84]
Sex (1 = Female)	2.308	3.365	.048	0.69	.494	[-4.33, 8.94]
Education	-0.496	2.716	-.014	-0.18	.855	[-5.85, 4.86]

Note. N = 208.

Table S7. Multiple Linear Regression Predicting Depressive Symptoms From ADHD Severity and Covariates

Predictor	B	SE	β	t	p	95% CI for B
ADHD Severity	2.955	1.241	.171	2.38	.018	[0.51, 5.40]
Age	0.155	0.171	.068	0.91	.366	[-0.18, 0.49]
Sex (1 = Female)	4.999	1.588	.226	3.15	.002	[1.87, 8.13]
Education	-1.533	1.275	-.094	-1.20	.231	[-4.05, 0.98]

Note. N = 191.

Table S8. Multiple Linear Regression Predicting State Anxiety From ADHD Severity and Covariates

Predictor	B	SE	β	t	p	95% CI for B
ADHD Severity	2.440	1.454	.124	1.68	.095	[-0.43, 5.31]
Age	0.133	0.199	.051	0.67	.506	[-0.26, 0.53]
Sex (1 = Female)	3.491	1.849	.139	1.89	.061	[-0.16, 7.14]
Education	-0.866	1.481	-.047	-0.59	.559	[-3.79, 2.06]

Note. N = 193.

Table S9. Multiple Linear Regression Predicting Trait Anxiety From ADHD Severity and Covariates

Predictor	B	SE	β	t	p	95% CI for B
ADHD Severity	2.386	1.190	.147	2.01	.046	[0.04, 4.73]
Age	0.199	0.163	.093	1.22	.223	[-0.12, 0.52]
Sex (1 = Female)	2.528	1.514	.122	1.67	.097	[-0.46, 5.51]
Education	-0.962	1.213	-.063	-0.79	.429	[-3.35, 1.43]

Note. N = 193.

Table S10. Multiple Linear Regression Predicting Problematic Internet Use (IAT Total) From ADHD Severity and Covariates

Predictor	B	SE	β	t	p	95% CI for B
ADHD Severity	4.545	1.842	.176	2.47	.014	[0.91, 8.18]
Age	0.264	0.236	.084	1.12	.265	[-0.20, 0.73]
Sex (1 = Female)	-3.225	2.217	-.104	-1.46	.147	[-7.60, 1.15]
Education	-1.126	1.758	-.050	-0.64	.523	[-4.59, 2.34]

Note. N = 200. IAT = Internet Addiction Test.

Table S11. Logistic Regression Predicting Sleep Disturbance From ADHD Severity and Covariates

Predictor	B	SE	Wald	p	OR	95% CI for OR
ADHD Severity	0.315	0.264	1.42	.233	1.37	[0.82, 2.30]
Age	0.066	0.037	3.15	.076	1.07	[0.99, 1.15]
Sex (1 = Female)	0.186	0.325	0.33	.567	1.21	[0.64, 2.28]
Education	-0.543	0.282	3.71	.054	0.58	[0.34, 1.01]

Note. N = 218.

Table S12. Multiple Linear Regression Predicting Duration of Untreated Illness (DUI) From ADHD Severity and Covariates

Predictor	B	SE	β	t	p	95% CI for B
ADHD Severity	0.082	0.718	.007	0.11	.910	[-1.34, 1.51]
Age	1.178	0.103	.798	11.43	< .001	[0.97, 1.38]
Sex (1 = Female)	0.549	0.872	.041	0.63	.530	[-1.18, 2.28]
Education	-0.245	0.777	-.023	-0.32	.753	[-1.79, 1.30]

Note. N = 97. DUI = Duration of Untreated Illness.

Table S13. Logistic Regression Predicting Substance Use Disorder From ADHD Severity and Covariates

Predictor	B	SE	Wald	p	OR	95% CI for OR
ADHD Severity	0.933	0.296	9.95	.002	2.54	[1.42, 4.54]
Age	0.006	0.036	0.03	.866	1.01	[0.94, 1.08]
Sex (1 = Female)	-0.593	0.356	2.77	.096	0.55	[0.28, 1.11]
Education	-0.882	0.277	10.15	.001	0.41	[0.24, 0.71]

Note. N = 219.

Table S14. Logistic Regression Predicting Mood Disorder From ADHD Severity and Covariates

Predictor	B	SE	Wald	p	OR	95% CI for OR
ADHD Severity	0.599	0.326	3.39	.066	1.82	[0.96, 3.45]
Age	-0.009	0.041	0.05	.832	0.99	[0.92, 1.07]
Sex (1 = Female)	-0.189	0.396	0.23	.632	0.83	[0.38, 1.80]
Education	-0.062	0.310	0.04	.841	0.94	[0.51, 1.73]

Note. N = 219.

Table S15. Logistic Regression Predicting Anxiety Disorder From ADHD Severity and Covariates

Predictor	B	SE	Wald	p	OR	95% CI for OR
ADHD Severity	-0.594	0.265	5.00	.025	0.55	[0.33, 0.93]
Age	0.025	0.035	0.53	.468	1.03	[0.96, 1.10]
Sex (1 = Female)	-0.324	0.323	1.01	.315	0.72	[0.38, 1.36]
Education	0.668	0.275	5.89	.015	1.95	[1.14, 3.35]

Note. N = 219.

Table S16. Logistic Regression Predicting Other Psychiatric Disorders From ADHD Severity and Covariates

Predictor	B	SE	Wald	p	OR	95% CI for OR
ADHD Severity	0.644	0.411	2.45	.118	1.90	[0.85, 4.26]
Age	0.097	0.049	3.85	.050	1.10	[1.00, 1.21]
Sex (1 = Female)	0.599	0.488	1.51	.220	1.82	[0.70, 4.74]
Education	-0.408	0.364	1.26	.262	0.67	[0.33, 1.36]

Note. N = 219.

Table S17. Logistic Regression Predicting Autism Spectrum Disorder From ADHD Severity and Covariates

Predictor	B	SE	Wald	p	OR	95% CI for OR
ADHD Severity	-0.433	0.409	1.12	.290	0.65	[0.29, 1.45]
Age	-0.016	0.055	0.09	.769	0.98	[0.88, 1.10]
Sex (1 = Female)	0.438	0.498	0.78	.379	1.55	[0.58, 4.11]
Education	-0.816	0.393	4.32	.038	0.44	[0.21, 0.95]

Note. N = 219.

Table S18. Association Between ADHD Presentation and Socio-Demographic Variables

Variable	Test	Statistic	df	p	Effect Size
Age	F	0.34	1, 211	.559	$\eta^2 = .002$
Sex	χ^2	0.48	1	.487	V = .047
Education	χ^2	4.70	3	.195	V = .148

Note. N ranges from 215 to 216 due to missing data.

Table S19. Logistic Regression Predicting Family Psychiatric History From ADHD Presentation

Predictor	B	SE	Wald	p	OR	95% CI for OR
ADHD Presentation	0.52	0.33	2.45	.117	1.69	[0.88, 3.24]
Age	0.04	0.03	1.66	.197	1.04	[0.98, 1.11]
Sex (1 = Female)	0.13	0.29	0.19	.665	1.13	[0.64, 2.00]
Educational level	-0.25	0.23	1.13	.288	0.78	[0.50, 1.23]

Table S20. Logistic Regression Predicting Family Psychiatric History From ADHD Severity

Predictor	B	SE	Wald	p	OR	95% CI for OR
ADHD Severity	0.64	0.24	7.13	.008	1.90	[1.19, 3.04]
Age	0.04	0.03	1.52	.217	1.04	[0.98, 1.11]
Sex	0.07	0.29	0.05	.820	1.07	[0.61, 1.88]
Educational level	-0.18	0.24	0.60	.438	0.83	[0.52, 1.32]

Table S21. Logistic Regression Predicting Family ADHD History From ADHD Presentation

Predictor	B	SE	Wald	p	OR	95% CI for OR
ADHD Presentation	0.25	0.48	0.27	.606	1.28	[0.50, 3.26]
Age	0.03	0.04	0.54	.462	1.03	[0.95, 1.12]
Sex	1.70	0.41	17.18	< .001	5.45	[2.44, 12.14]
Educational level	-0.03	0.31	0.01	.935	0.98	[0.54, 1.78]

Table S22. Logistic Regression Predicting Family ADHD History From ADHD Severity

Predictor	B	SE	Wald	p	OR	95% CI for OR
ADHD Severity	0.01	0.31	0.00	.976	1.01	[0.55, 1.84]
Age	0.04	0.04	0.71	.400	1.04	[0.95, 1.13]
Sex	1.70	0.41	17.29	< .001	5.47	[2.46, 12.19]
Educational level	-0.06	0.31	0.04	.836	0.94	[0.51, 1.72]

Table S23. Logistic Regression Predicting Current Psychotherapy From ADHD Presentation

Predictor	B	SE	Wald	p	OR	95% CI for OR
ADHD Presentation	0.31	0.34	0.81	.367	1.36	[0.70, 2.67]
Age	0.01	0.03	0.18	.675	1.01	[0.95, 1.08]
Sex	0.41	0.29	2.02	.155	1.51	[0.86, 2.65]
Educational level	0.38	0.23	2.73	.098	1.47	[0.93, 2.31]

Table S24. Logistic Regression Predicting Current Psychotherapy From ADHD Severity

Predictor	B	SE	Wald	p	OR	95% CI for OR
ADHD Severity	0.20	0.23	0.71	.398	1.22	[0.77, 1.93]
Age	0.01	0.03	0.11	.737	1.01	[0.95, 1.07]
Sex	0.32	0.29	1.27	.260	1.38	[0.79, 2.41]
Educational level	0.42	0.24	3.22	.073	1.53	[0.96, 2.43]

Table S25. Logistic Regression Predicting Organic Comorbidity From ADHD Presentation

Predictor	B	SE	Wald	p	OR	95% CI for OR
ADHD Presentation	0.02	0.37	0.00	.961	1.02	[0.50, 2.09]
Age	0.03	0.03	0.74	.391	1.03	[0.96, 1.10]
Sex (1 = Female)	0.81	0.31	6.80	.009	2.24	[1.22, 4.11]
Educational level	-0.23	0.24	0.87	.352	0.80	[0.49, 1.29]

Table S26. Logistic Regression Predicting Organic Comorbidity From ADHD Severity

Predictor	B	SE	Wald	p	OR	95% CI for OR
ADHD Severity	0.11	0.25	0.20	.658	1.12	[0.68, 1.83]
Age	0.03	0.03	0.82	.364	1.03	[0.97, 1.10]
Sex	0.79	0.31	6.59	.010	2.21	[1.21, 4.03]
Educational level	-0.23	0.25	0.87	.352	0.79	[0.49, 1.29]

Table S27. Logistic Regression Predicting Organic Comorbidity Including Presentation × Sex Interaction

Predictor	B	SE	Wald	p	OR	95% CI for OR
ADHD Presentation	0.29	0.52	0.31	.579	1.33	[0.48, 3.66]
Sex	1.26	0.67	3.57	.059	3.53	[0.95, 13.04]
Presentation × Sex	-0.58	0.74	0.60	.440	0.56	[0.13, 2.42]
Age	0.03	0.03	0.82	.365	1.03	[0.97, 1.10]
Educational level	-0.23	0.24	0.91	.341	0.79	[0.49, 1.28]

Table S28. ANCOVA Predicting Quality of Life From ADHD Presentation

Predictor	F	df	p	Partial η^2
ADHD Presentation	0.49	1, 189	.485	.003
Age	2.00	1, 189	.158	.010
Sex	8.85	1, 189	.003	.045
Education	0.70	1, 189	.404	.004

Table S29. Multiple Linear Regression Predicting Quality of Life From ADHD Severity

Predictor	B	SE	β	t	p
ADHD Severity	-4.17	1.78	-.163	-2.34	.020
Age	-0.32	0.23	-.103	-1.41	.160
Sex (1 = Female)	-6.70	2.15	-.218	-3.12	.002
Education	-2.28	1.70	-.102	-1.34	.180

Table S30. Logistic Regression Predicting Hospitalization

Predictor	B	OR	p
DERS	0.026	1.027	.021
ZUNG	-0.288	0.75	.295
STAI-State	-0.108	0.90	.684
IAT	0.009	1.01	.589
BIS	0.019	1.02	.217
Age	-0.011	0.99	.828
Sex	0.678	1.97	.219
Education	-1.055	0.35	.004

Table S31. Logistic Regression Predicting Hospitalization From ADHD Severity and Presentation

Predictor	B	SE	Wald	p	OR	95% CI
ADHD Severity	0.534	0.361	2.19	.139	1.71	[0.84, 3.46]
ADHD Presentation (Combined vs Inattentive)	1.257	0.774	2.64	.104	3.52	[0.77, 16.04]
Age	0.017	0.043	0.15	.695	1.02	[0.94, 1.11]
Sex (1 = Female)	0.315	0.419	0.57	.452	1.37	[0.60, 3.12]
Education	-0.972	0.324	8.98	.003	0.38	[0.20, 0.72]

Table S32. ANCOVA Predicting Duration of Untreated Illness From ADHD Presentation

Predictor	F	df	p	Partial η²
ADHD Presentation	1.17	1, 90	.282	.013
Age	144.00	1, 90	< .001	.615
Sex	0.01	1, 90	.940	.000
Education	0.12	1, 90	.731	.001

Note. $N = 95$.

Table S33. Logistic Regression Predicting Current Pharmacological Treatment From ADHD Presentation

Predictor	B	SE	Wald	p	OR	95% CI for OR
ADHD Presentation (Combined vs Inattentive)	0.558	0.339	2.71	.100	1.75	[0.90, 3.40]
Age	-0.029	0.032	0.81	.368	0.97	[0.91, 1.03]
Sex (1 = Female)	-0.202	0.298	0.46	.498	0.82	[0.46, 1.47]
Education	-0.489	0.244	4.03	.045	0.61	[0.38, 0.99]

Note. $N = 215$.

Table S34. Logistic Regression Predicting Current Pharmacological Treatment From ADHD Severity

Predictor	B	SE	Wald	p	OR	95% CI for OR
ADHD Severity	0.307	0.240	1.63	.201	1.36	[0.85, 2.18]
Age	-0.028	0.032	0.75	.387	0.97	[0.91, 1.04]
Sex (1 = Female)	-0.244	0.293	0.69	.405	0.78	[0.44, 1.39]
Education	-0.436	0.244	3.20	.074	0.65	[0.40, 1.04]

Note. N = 219.

Table S35. STROBE Statement — Checklist of Items for Cross-Sectional Studies

Section/Topic	Item	Recommendation	Reported in (section)
Title and abstract	1	(a) Indicate the study's design with a commonly used term in the title or the abstract; (b) provide an informative and balanced summary of what was done and what was found.	Title; Abstract ("retrospective, cross-sectional study")
**Introduction**			
Background/rationale	2	Explain the scientific background and rationale for the investigation being reported.	Section 1, paragraphs 1–6
Objectives	3	State specific objectives, including any prespecified hypotheses.	Section 1, last paragraph; Section 2.4 (inferential hierarchy)
**Methods**			
Study design	4	Present key elements of study design early in the paper.	Section 2.1
Setting	5	Describe the setting, locations, and relevant dates, including periods of recruitment, exposure, follow-up, and data collection.	Section 2.1 (setting, recruitment period); Section 2.3 (timing of assessments)
Participants	6	(a) Give the eligibility criteria, and the sources and methods of selection of participants.	Section 2.2
Variables	7	Clearly define all outcomes, exposures, predictors, potential confounders, and effect modifiers. Give diagnostic criteria, if applicable.	Sections 2.2 (ADHD diagnostic procedure) and 2.3 (severity, psychometric measures, comorbidities, hospitalization, DUI, treatment)
Data sources/measurement	8*	For each variable of interest, give sources of data and details of methods of assessment (measurement). Describe comparability of assessment methods if there is more than one group.	Sections 2.2–2.3
Bias	9	Describe any efforts to address potential sources of bias.	Section 2.4 (missing data, multiplicity, penalized regression); Section 4.8

Section/Topic	Item	Recommendation	Reported in (section)
Study size	10	Explain how the study size was arrived at.	Section 2.2 (all consecutive eligible patients in the study period; no a priori sample size calculation)
Quantitative variables	11	Explain how quantitative variables were handled in the analyses. If applicable, describe which groupings were chosen and why.	Sections 2.3–2.4 (coding of severity and education; sensitivity analyses)
Statistical methods	12	(a) Describe all statistical methods, including those used to control for confounding; (b) describe any methods used to examine subgroups and interactions; (c) explain how missing data were addressed; (d) if applicable, describe analytical methods taking account of sampling strategy; (e) describe any sensitivity analyses.	Section 2.4: (a) covariate adjustment; (b) presentation × sex interaction; (c) complete-case analysis and completer comparison; (d) not applicable; (e) dummy-coded severity/education, Firth regression, FDR
**Results**			
Participants	13*	(a) Report numbers of individuals at each stage of study; (b) give reasons for non-participation at each stage; (c) consider use of a flow diagram.	Section 2.2 (flow of participants); Table 1
Descriptive data	14*	(a) Give characteristics of study participants and information on exposures and potential confounders; (b) indicate number of participants with missing data for each variable of interest.	Table 1; Section 3.1
Outcome data	15*	Report numbers of outcome events or summary measures.	Table 1; Sections 3.3, 3.5, 3.7
Main results	16	(a) Give unadjusted estimates and, if applicable, confounder-adjusted estimates and their precision (e.g., 95% CI); make clear which confounders were adjusted for and why; (b) report category boundaries when continuous variables were categorized; (c) if relevant, consider translating estimates of relative risk into absolute risk.	Sections 3.2–3.7; Tables 2–7; Supplementary Tables S1–S34
Other analyses	17	Report other analyses done—e.g., analyses of subgroups and interactions, and sensitivity analyses.	Sections 3.3–3.7; Table 8; Supplementary Tables S27, S36, S37
**Discussion**			
Key results	18	Summarise key results with reference to study objectives.	Section 4 (opening paragraph); Section 5
Limitations	19	Discuss limitations of the study, taking into account sources of potential bias or imprecision. Discuss both direction and magnitude of any potential bias.	Section 4.8
Interpretation	20	Give a cautious overall interpretation of results considering objectives, limitations, multiplicity of analyses, results from similar studies, and other relevant evidence.	Sections 4.1–4.7; Section 5
Generalisability	21	Discuss the generalisability (external validity) of the study results.	Section 4.8 (second paragraph)
**Other information**			
Funding	22	Give the source of funding and the role of the funders for the present study and, if applicable, for the original study on which the present article is based.	Funding statement (no external funding)

Note. Adapted from von Elm et al. (2007). Locations refer to sections of the revised manuscript.

Table S36. Comparison of Participants With Complete Versus Incomplete Psychometric Data

	Incomplete data (n = 133)	Complete data (n = 86)	Test statistic	p
Age, M (SD)	26.08 (4.95)	26.29 (4.81)	t(217) = −0.31	.759

	Incomplete data (n = 133)	Complete data (n = 86)	Test statistic	p
**Sex, n (%)**			$\chi^2(1) = 11.63$	.001
Male	89 (66.9)	37 (43.5)		
Female	44 (33.1)	48 (56.5)		
**Educational level, n (%)**			$\chi^2(2) = 0.85$	.654
Low education	23 (17.3)	11 (12.8)		
High school	72 (54.1)	48 (55.8)		
University degree	38 (28.6)	27 (31.4)		
**ADHD presentation, n (%)**			$\chi^2(1) = 0.04$	.852
Combined	104 (78.2)	64 (77.1)		
Inattentive	29 (21.8)	19 (22.9)		
**ADHD severity, n (%)**			$\chi^2(2) = 1.55$	.460
Mild	14 (10.5)	14 (16.3)		
Moderate	83 (62.4)	50 (58.1)		
Severe	36 (27.1)	22 (25.6)		

Note. Values are M (SD) for age and n (%) for categorical variables; percentages are calculated within completeness groups. Age was compared with an independent-samples t test; categorical variables with Pearson chi-square tests.

Table S37. Sensitivity Analyses of ADHD Severity and Educational Level Using Ordinal and Dummy Coding

Outcome	Predictor / contrast	B	SE	β / OR	95% CI	p
DERS	ADHD severity, linear trend	9.44	2.69	β = .245	[4.12, 14.75]	.001
	Moderate vs. mild	3.50	4.95	β = .072	[−6.26, 13.26]	.480
	Severe vs. mild	16.82	5.56	β = .313	[5.85, 27.79]	.003
	Education, linear trend	−1.03	2.81	β = −.028	[−6.58, 4.51]	.713
	High school vs. low education	−7.45	4.85	β = −.154	[−17.00, 2.11]	.126
	University vs. low education	−3.76	5.70	β = −.071	[−14.99, 7.47]	.510
Current SUD	ADHD severity, linear trend	—	—	OR = 2.50	[1.40, 4.46]	.002
	Moderate vs. mild	—	—	OR = 1.91	[0.52, 7.09]	.331
	Severe vs. mild	—	—	OR = 5.24	[1.35, 20.41]	.017
	Education, linear trend	—	—	OR = 0.39	[0.22, 0.69]	.001
	High school vs. low education	—	—	OR = 0.26	[0.11, 0.61]	.002
	University vs. low education	—	—	OR = 0.17	[0.06, 0.50]	.001

Note. All models were adjusted for age and sex; models of ADHD severity were also adjusted for educational level, and models of educational level for ADHD severity. Mild severity and low education

were the reference categories in dummy-coded models. Odds ratios are expressed for the presence of current SUD.

Table S38. Firth Penalized Logistic Regression for Psychiatric Hospitalization

Predictor	B	SE	OR	95% CI for OR	p
DERS	0.023	0.011	1.02	[1.00, 1.05]	.025
ZUNG	−0.269	0.263	0.76	[0.46, 1.28]	.306
STAI-State	−0.092	0.249	0.91	[0.56, 1.49]	.712
IAT total	0.008	0.016	1.01	[0.98, 1.04]	.625
BIS-11	0.017	0.015	1.02	[0.99, 1.05]	.237
Age	−0.007	0.049	0.99	[0.90, 1.09]	.882
Sex	0.669	0.524	1.95	[0.70, 5.45]	.202
Educational level	−1.030	0.368	0.36	[0.17, 0.73]	.004

Note. N = 172; hospitalization events = 24; non-events = 148; events per predictor = 3.0. Estimates were obtained with Firth's bias-reduced penalized-likelihood logistic regression; odds ratios are expressed for the presence of psychiatric hospitalization.
